# Experimental Setups for In Vitro Studies on Radon Exposure in Mammalian Cells—A Critical Overview

**DOI:** 10.3390/ijerph20095670

**Published:** 2023-04-27

**Authors:** Andreas Maier, Tarryn Bailey, Annika Hinrichs, Sylvie Lerchl, Richard T. Newman, Claudia Fournier, Charlot Vandevoorde

**Affiliations:** 1 Biophysics Department, GSI Helmholtzzentrum für Schwerionenforschung GmbH, 64291 Darmstadt, Germany; 2 Department of Physics, Stellenbosch University, Stellenbosch, Cape Town 7600, South Africa; 3 Radiation Biophysics Division, Separated Sector Cyclotron Laboratory, NRF-iThemba LABS, Cape Town 7129, South Africa; 4 Physics Department, Goethe University Frankfurt am Main, 60438 Frankfurt am Main, Germany

**Keywords:** radon exposure, molecular mechanisms, radon chamber, radon analogue, radiobiology, in vitro experiments, alpha particles, DNA damage

## Abstract

Naturally occurring radon and its short lived progeny are the second leading cause of lung cancer after smoking, and the main risk factor for non-smokers. The radon progeny, mainly Polonium-218 (^218^Po) and Polonium-214 (^214^Po), are responsible for the highest dose deposition in the bronchial epithelium via alpha-decay. These alpha-particles release a large amount of energy over a short penetration range, which results in severe and complex DNA damage. In order to unravel the underlying biological mechanisms which are triggered by this complex DNA damage and eventually give rise to carcinogenesis, in vitro radiobiology experiments on mammalian cells have been performed using radon exposure setups, or radon analogues, which mimic alpha-particle exposure. This review provides an overview of the different experimental setups, which have been developed and used over the past decades for in vitro radon experiments. In order to guarantee reliable results, the design and dosimetry of these setups require careful consideration, which will be emphasized in this work. Results of these in vitro experiments, particularly on bronchial epithelial cells, can provide valuable information on biomarkers, which can assist to identify exposures, as well as to study the effects of localized high dose depositions and the heterogeneous dose distribution of radon.

## 1. Introduction

Radon is the second leading cause of lung cancer worldwide, after tobacco smoking, and estimated by the World Health Organization to cause between 3% to 14% of all lung cancers, depending on the country [1]. It is a natural, colorless, tasteless and odorless radioactive noble gas which is released in the natural occurring decay of ^235^U, ^232^Th and ^238^U, which are present in rock and soil [2]. The three corresponding isotopes are ^219^Rn (actinon, T_1/2_ = 3.96 s), ^220^Rn (thoron, T_1/2_ = 55.6 s) and ^222^Rn (radon, T_1/2_ = 3.82 days), respectively. However, the majority of the radioactivity in the atmosphere at sea level is attributable to the isotopes ^220^Rn and ^222^Rn, of which ^222^Rn is the most abundant of all radon isotopes [3]. Comparing the potential alpha-energy concentration of radon and thoron reveals a higher value for thoron, but due to its short half-life it is only able to diffuse a few centimeters (cm) between formation and decay. As a result, the thoron levels in the air are lower compared to radon, which can diffuse more than 1 m in soil, for example, in workplaces [3].

In general, it is anticipated that low outdoor radon concentrations do not represent a significant health risk to the public, since radon is quickly diluted to low concentrations in the air after exhaling from the ground [4]. The situation indoors is quite different, where radon can rapidly accumulate in poorly ventilated buildings, especially in basements, when it seeps in through cracks in the floor and foundations [1]. As a result, many countries have established regulations and recommendations on radon concentration levels in workspaces and homes [5]. 

Because the residential radon exposure can contribute to the onset of lung cancer in the overall population, the perception and communication of radon risks continues to receive a lot of research interest [6,7,8]. In addition, radon concentrations vary spatially depending on different geology factors and are particularly elevated in areas with phosphate rocks, metamorphic rocks and uranium ores [9]. Additionally, anthropogenic causes such as water supply [10], building materials [11] and soil sealing [12] can lead to increased radon activity concentrations. Particularly in uranium mines, the radon gas can reach high concentrations, and as a result, several case-control studies have been carried out to determine the correlation between radon levels and the probability of lung cancer development in uranium miners [13,14,15,16]. 

The International Agency for Research on Cancer (IARC) listed radon and its decay products as Group 1 carcinogenic agents back in 1988 [17]. Over the years, a large pool of strong and complementary evidence has been collected by multiple research groups in several countries which confirm the increased risk of lung cancer from the cumulative exposure to radon and its progeny via inhalation [18,19,20,21,22,23,24]. A collaborative analysis from several European case-control studies have provided enough evidence to conclude that radon concentrations in ordinary homes are causing lung cancer, with an estimated increased risk of 16% per 100 Bq/m^3^ [18]. 

While the respiratory tract remains the primary target for radon-induced carcinogenesis, especially due to the deposition of radioactive progeny, radon gas can be dissolved in the blood and further distributed and deposited in various tissues and organs throughout the body. The radiation doses that reach these tissues and organs will be several times lower than the dose received by the lungs, but they might contribute to an increased risk of non-pulmonary cancers [25]. 

In addition, a recent investigation suggests that radioactive radon progeny might be retained in the body for longer periods than expected and therefore increase the absorbed dose [26]. As a result, a growing number of studies investigate whether radon also contributes to carcinogenic effects in the skin, brain and central nervous system, stomach and leukemia [27,28,29,30,31,32,33]. However, a recent review clearly highlights the limited number and the heterogeneity of existing studies, which limits the statistical power to make sound conclusions on the causal association between radon exposure and non-pulmonary neoplasms [25]. 

On the one hand, the data available from epidemiological studies can be used to determine radon-related cancer risks, while on the other hand, it is also possible to perform risk assessments based on dose calculations [34,35]. When air containing radon gas and its short-lived radioactive progeny are inhaled, radon gas is, for the most part, exhaled before it decays. Directly after radon decays (<1 s), the progeny attaches to water vapor and trace gases, forming the so-called “unattached fraction”, with a size smaller than 5 nm. These might further attach to existing aerosol particles resulting in the “attached fraction” ranging from 10 nm to several μm [36,37]. Based on measurements of larger and medium sized particles and simulations, the deposition depends strongly on the progeny’s size. 

Next to the size distribution and fraction of radon progeny, the concentration of lung-deposited radon progeny will strongly depend on physical and behavioral characteristics which can affect the breathing rate of the individual being exposed [38]. It is difficult to measure the deposited activity in the respiratory system of the human being, and therefore, the absorbed doses are calculated from models which require input on the size distribution and activity concentration of unattached and attached fractions [39]. Simulation studies suggest that short lived progeny account for more than 95% of the total effective dose, while the radon gas itself contributes less than 5% [3], as only about 1% of the gas is absorbed by the blood [40]. 

When the short-lived radon progeny are deposited in the airway after their inhalation, they can deposit significant energy within the human respiratory tract as a result of alpha-decay [41]. In general, it is expected that the bronchial epithelium that lines the airways is the most exposed tissue during radon exposure [42]. In terms of radiation dose, ^218^Po (polonium-218, T_1/2_ = 3.097 min) and ^214^Po (polonium-214, T_1/2_ = 164.3 μs) are responsible for the highest dose deposition [2]. However, determining a reliable dose for radon exposure is complex as the distribution of radon progeny after inhalation in the lungs is non-uniform and the limited range of alpha-particles results in very localized energy depositions. Therefore, one of the main uncertainties in experimental radon studies is related to the dosimetry of internally deposited radon progeny, which is fundamental and essential to understand the biological observations. 

Since alpha-particles are responsible for a major part of the total absorbed dose in the lungs from radon exposure, approximately 97% according to simulations, they are the main focus of current and past radiobiology studies on the oncogenic effects of radon exposure [43,44]. Therefore, radon chambers or radon analogues, using radionuclides that emit alpha-particles in a similar energy range as the radon decay products, are commonly used to perform in vitro or in vivo experiments to evaluate the biological effects of radon exposure. 

These alpha-particles have high-Linear Energy Transfer (LET), which was calculated to be 166 keV/µm for an alpha-particle of 2.5 MeV. This is many orders of magnitude above the calculated LET of X-rays (250 kVp) of 2.0 keV/µm or Cobalt-60 gamma-rays (1.1–1.3 MeV) of 0.2 keV/µm [45]. An alpha-particle deposits a high amount of energy along a relatively short penetration range of 50–100 µm in soft tissue, resulting in severe and complex DNA damage, which is challenging for a cell to repair [46]. As a result, it has been postulated that a single alpha-particle traversing a cell can be sufficient to induce cell killing, although this statement has been challenged over the years [47,48]. 

In addition to cells that are directly hit by alpha-particles, there is also a growing number of studies that show biological effects in non-irradiated neighboring cells, caused by bystander effects via gap junction communication with exposed cells [49,50,51,52,53]. 

Recently, a multi-scaled Monte Carlo study on radon-induced cellular damage in the bronchial airway epithelium simulated the spatial distribution of ionizing events and estimated the damage distribution in the cellular DNA of six cell types in the lung epithelium [54]. The study showed that deep-seated nuclei were less prone to being hit, but the DNA damage from a single hit would be more severe. The latter assumption is based on the knowledge that slowing down of the penetrating alpha-particles, such as the 7.69 MeV alpha-particles emitted from ^214^Po, could reach those deep-seated cells and will have a higher LET due to the energy loss. The latter is directly linked with the biological effects that are induced, since a higher density of ionization events along the track of high-LET radiation will result in a higher probability to induce a biological effect compared to low-LET radiation [55].

As a result of the more lethal and complex DNA damage that is induced by high-LET radiation, its biological efficiency will be higher. The latter concept is defined as the relative biological effectiveness (RBE) of different radiation qualities. 

RBE is calculated as the absorbed dose of a reference radiation (e.g., 250 kVp X-rays, ^60^Co gamma-rays) required to produce a certain level of biological effect, divided by the absorbed dose of the test radiation (e.g., alpha-particles) required to induce the same level of biological effect. The value depends on several parameters, including the radiation quality, the biological effect under investigation, dose rate and cell type. While many studies have been conducted so far to determine the RBE of alpha-particles, there is a large divergence in reported RBE values. The latter is not surprising given the number of parameters that influence RBE, as well as the fact that the LET of alpha-particles strongly depends on their energy, which will differ for the different radon progeny. Therefore, it is of critical importance that the irradiation setup and dosimetry in experimental studies are well characterized. 

Due to the complexities associated with the dose distribution of radon progeny in the lungs and uncertainties in the absorbed dose, in vivo studies might be more appropriate to evaluate the biological effects of radon exposure. However, despite the shortcomings of in vitro studies, these experiments are still very useful to improve our understanding of the fundamental biological mechanisms that are responsible for the biological observations at the tissue and organism level [56]. 

In addition, the in vitro results can be used as input for mechanistic modelling to simulate biophysical processes and validate microdosimetric simulations [57]. Therefore, this current review provides an overview of the various in vitro irradiation setups, including radon chambers and radon analogues, that have been used by different research groups to expose mammalian cells to radon and alpha-particles with kinetic energies similar to those of radon and its progeny. To the best of our knowledge, this is the first time such a complete overview of in vitro studies has been compiled, which is complementary to previously published reviews which have provided an overview of in vivo, epidemiological and modeling studies [22,44,58,59]. It allows an in-depth comparison of the different biological observations that have been made so far under different radon exposure conditions. It provides the possibility to identify critical parameters and variables which might contribute to the large variation in reported biological effects in radon and radon analogue studies. 

## 2. In Vitro Studies on Mammalian Cells Exposed to Radon and Progeny

**Figure 1:**
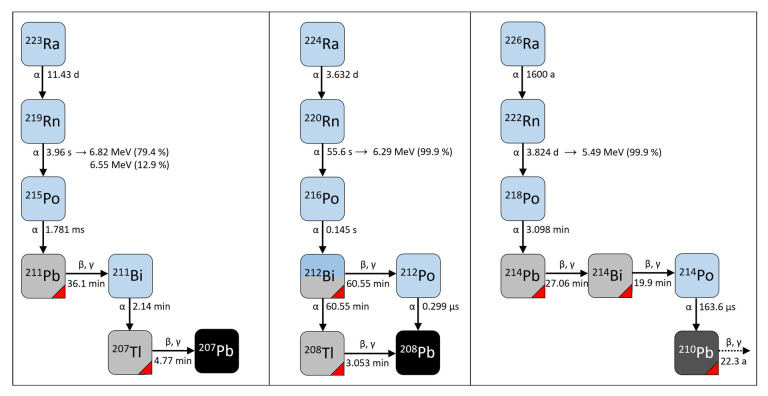
A schematic of the decay schemes for ^219^Rn, ^220^Rn and ^222^Rn. The blue and grey squares represent unstable isotopes that undergo the alpha- and beta-decay respectively, while the red corner refers to isotopes that also undergo gamma-decay. (adapted from [63]).

In order to elucidate the cellular effects of radon and its short-lived progeny, several research groups exposed mammalian cells in vitro by using specialized experimental setups (see Table 1 for an overview). The exposure to radon and its progeny results in a mixed radiation field, consisting of alpha-, beta- and gamma-radiation. The decay scheme of the different radon isotopes with their respective half-lives is represented in Figure 1.

This mixed radiation field, together with the fact that radon is an inert gas, brings an additional level of complexity to these kind of experiments, where not only the homogenous exposure of the cells is difficult, but also the dosimetry remains a very challenging task [60]. In addition, the short range of alpha-particles in the order of a few cm in the air and a few μm in water or similar materials makes it challenging to irradiate in vitro mammalian cells which are in the order of 10–100 µm and routinely grown in sterile plastic cell culture plates in the presence of a growth medium. Due to the very short range of alpha-particles, the alpha-decay would need to take place in close proximity to the cells in order to cause damage. This means that for cells covered with medium, radon needs to be dissolved beforehand. This will reduce the applied dose significantly, as the solubility of radon in isotone solution, which is similar to cell culture medium, is low [61,62]. In the following paragraphs, various irradiation methods enabling exposure of cells to radon and its progeny are described, together with the dosimetry as well as the main radiobiological findings.

In general, there are two main variations in experimental in vitro setups to perform irradiation with radon and progeny. While some studies expose adherent mammalian cells directly to radon gas, others irradiate cells in suspension by using radon-saturated medium or the addition of a ^223^Ra-dichloride (^223^RaCl_2_) solution to the medium.

### 2.1. Radon Exposure Setups for Adherent Mammalian Cell Cultures

Exposing adherent cells in vitro to radon needs to fulfil radiation protection requirements. In order to guarantee the operator’s safety during experiments, the studies are performed within airtight setups, such as chambers where radon can be introduced by flushing sources with air at the beginning of the exposure [64,65,66], continuously throughout the exposure time [67], or in complete incubators containing radon gas [68]. These exposure units provide the opportunity to regulate temperature [67], humidity and CO_2_-concentration [64,65]. 

The latter is particularly important for protracted low-dose irradiations, which require longer exposure times, while general cell viability needs to be maintained by providing optimal cell culture conditions. Additionally, medium supply needs to be guaranteed to prevent the cells from drying out and, simultaneously, the direct contact of the cells with radon and progeny is favorable in order to perform dosimetry. The latter can be achieved by performing the radon exposure in so-called Transwell^®^ (Corning Costar Corporation, Corning, NY, USA) plates with cells growing on a polyester membrane floating on top of the culture medium [64]. In principle, it is also possible to transfer this technique to non-adherent cells, such as human peripheral blood lymphocytes (HPBLs), by pre-coating Transwell^®^ inserts overnight with poly-l-lysine and poly-ornithine and therefore allow adhesion of the non-adherent cells [66,69]. Other groups removed the medium above the cells completely [67] or minimized the amount of culture medium (750 μL) in the culture plates where cells were seeded [70], resulting in a minimal diffusion barrier. 

The study of Loiselle et al. used a setup which was constructed to enable long term exposure of adherent human lung epithelial cells to low radon activity concentrations (38 Bq/m^3^) [68]. During this experiment, the cells were exposed in 100 mm^3^ culture plates with the lid on in the incubator for up to four and a half months, together with a batholith hematite granite (Standard 59033) rock source of 5 kg containing minimal amounts of uranium and thorium in the order of parts per million. Considering that radon is denser than air, the rock source was placed in the upper shelf, while the cells were located on the lower shelf.

### 2.2. Radon Exposure Setups for Mammalian Cells in Suspension

In contrast to the exposure of adherent cells, many experimental setups were designed to irradiate cells in suspension and expose, for instance, whole blood samples. Most researchers adapted their setups in order to achieve a steady state between radon and progeny inside the medium by waiting at least 4 h before the cells were introduced for exposure. 

In an early study, Jostes et al. developed a method to irradiate cells in suspension in multiple steps. During the first step, a simpler irradiation scheme starting from ^212^Bi was used, where, independent of the decay of ^212^Bi, only one alpha-particle is emitted during decay to ^208^Pb [60]. ^212^Bi has a probability of 35.94% to decay to ^208^Tl with the emission of an alpha-particle and a probability of 64.06% to decay into ^212^Po via beta- and gamma-decay. In order to achieve this, a system consisting of an existing cation-exchange column was used, containing ^224^Ra [71]. Through further radioactive decay, ^212^Bi is generated inside this column and can be eluted with 1 mL of hydrogen iodide solution (HI) due to differences in affinity to the cation-exchange column between the occurring nuclides. Afterwards, the acidity level was adjusted to pH 5 with sodium acetate, and consequently, the cells were exposed in suspension with medium and the radioactive solutions. 

A second setup was implemented by the same group using a radium source and bubbling radon gas through the culture medium for 8 min in a spinner flask. After achieving a steady state, cells were added and exposed to low levels of radon and progeny for 3–19 h. 

In their third assembly, a ^226^RaCl_2_ source was used and radon gas was passed overnight over the surface of the culture medium, which was stirred continuously in a spinner flask. Additional CO_2_ was added to the radon/air mixture to adjust the pH to about 7.4. Cells were added after 4–18 h and exposed for 2–4 h to high levels of radon. An analogue procedure was used by Wolff et al. [72], passing radon originating from a ^226^RaCl_2_ source together with CO_2_ over the cell culture medium within a spinner flask for 4 h. Afterwards, cells were injected using a syringe and kept in spinner suspension during the entire exposure.

Bakale et al. developed another experimental setup, including a radium (^226^Ra) source to generate radon gas [73], which can be transferred to a spinner flask containing cell culture medium using a syringe and stirring the system for 4 h. A revised protocol for a higher sample throughput was additionally presented by inserting needles into spinner flasks and connecting several flasks in a row after the radon loading was already performed. Continuous circulation of radon, which was enabled by a pump and a larger number of experiments, is possible in parallel. 

Similar to this first setup, Hamza et al., 2008 developed a system in order to expose whole blood [74]. Radon originating from the radon source (Model RN-1025, Pylon Electronics Inc., Ottawa, ON, Canada) was injected into a bottle containing the blood sample. Keeping this inside an incubator in a roller platform for 3 h, the authors assumed uniform irradiation. In contrast to all other setups to irradiate cells in suspension, Hamza et al. did not wait for establishment of a steady state between radon and progeny.

In contrast, Schumann et al., 2018 based their experimental design on a clinically used radium isotope and mixed 1 mL of a ^223^Ra-Cl_2_ solution (Xofigo^®^, Bayer Vital GmbH, Leverkusen, Germany) diluted in PBS with 3.5 mL of blood samples [75]. Depending on the mixture of ^223^Ra-Cl_2_ with PBS, different activity concentrations were achieved, and once the steady state of radon and progeny was established, the cells were added and consequently exposed.

### 2.3. Dosimetric Considerations for Cell Exposures Using Radon Exposure Setups

Exposure of cells in vitro to radon and its progeny does not only need a thorough evaluation of the biological effects in order to elucidate potential risks and beneficial effects, but in order to classify these, appropriate dosimetry is mandatory. Therefore, different exposure parameters have to be monitored during the cell exposures and additional parameters should be calculated afterwards.

One main exposure parameter is radon activity concentration (c(Rn)), which can be determined using commercially available measurement devices. One group of devices uses alpha-spectrometry in order to measure the alpha-emitting progeny of radon (^220^Rn: ^216^Po, ^212^Po; ^222^Rn: ^218^Po, ^214^Po). These devices do not estimate radon activity for ^219^Rn due to its short half-life of 3.96 s (Figure 1). Radon-containing air is directed into the measurement device and alpha-particles are detected with a solid state alpha detector, enabling the distinction between the different nuclides based on their specific decay energy. The calculated activity of radon progeny then allows an extrapolation of radon activity concentration via calibration factors. In the irradiation setups described in this section, a RAD 7 monitor (Durridge Company Inc., Billerica, MA, USA) [64,69] and RTM 1688-2 (Sarad GmbH, Dresden, Germany) [65] were used. 

Another possibility is scintillation counters, of which there are several varieties. For the measurement of ^218^Po and ^214^Po, the passive AB5 radiation monitor (Pylon Electronics Inc., Ottawa, OTT, Canada) [68] or a Lucas cell [74] was used. These instruments contain a scintillator, which emits photons when an alpha-particle hits it. Through the connection to a photomultiplier, the signal is increased and detected afterwards, as the multiplied signal is proportional to the ionizing radiation energy. First, the activity of the detected progeny can be calculated and afterwards radon activity is extrapolated. 

Another method to determine the radon activity concentration focusses on the detection of the gamma-emitting progeny. Jostes et al. not only used a liquid scintillation counter LS 5801 to determine activity of alpha-emitting progeny (^218^Po, ^214^Po) (Beckman, Fullerton, CA, USA), but additionally used a NaI detector to measure gamma-emitting progeny [60,73]. 

Another measurement method is to use high purity germanium (HPGe) detectors. These types of detectors are semiconductor diodes producing charge carriers (holes and electrons) when photons interact with the germanium. The emerging charge is proportional to the energy deposited in the detector by radiation and can therefore be measured. Through decay correction of the measured gamma-emission (^223^Ra, ^219^Rn, ^211^Bi), activity can be quantified [75].

The ratio of radon compared to its alpha-emitting progeny (e.g., ^222^Rn: ^218^Po: ^214^Po) is an important parameter in order to calculate the potential alpha-energy concentration (PAEC). This concept is used in radiation protection considering higher decay energies of alpha-emitters (Figure 1) and describes the total alpha-energy emitted during the complete decay to the long-living Pb-nuclide. 

After the establishment of a radioactive equilibrium, Jostes et al. used the above-mentioned liquid scintillation counter LS 5801 (Beckman, Fullerton, CA, USA) and the NaI detector to measure the activity of alpha- and gamma-emitting progeny in the medium, respectively [60]. By solving the Bateman equations considering growth and decay of radon and progeny, the respective activity was calculated [76]. This equation is used in order to determine the decay (*N*) if several decays occur in the observed chain and can be written as follows:

$$N_{n}\left(t\right)=N_{1}\left(0\right)\left(\prod_{i=1}^{n-1}\lambda_{i}\right)\sum_{i=1}^{n}\frac{e^{-\lambda_{i}t}}{\prod_{j=1,j\neq i}^{n}\left(\lambda_{j}-\lambda_{i}\right)}$$

with *t* = time and *λ* = decay constant. The same method was applied by Bakale et al. to calculate progeny activity, and together with the known alpha- and beta-particle energies, the dose rate was calculated [73].

In contrast to this, Petitot et al. collected progeny on a membrane filter in the exposure chamber and measured independent alpha-particle counts (^218^Po, ^214^Po) of these filters at three different points [64]. The authors describe a modification of a previously published method to calculate the activity of radon progeny [77,78]. Moreover, they investigated the aerosol concentration and size distribution within their setup using a diffusion battery (model 3042A TSI Incorporated, St. Paul, MN, USA) associated with a condensation nuclei counter (model 3025A TSI Incorporated, St. Paul, MN, USA), revealing a unimodal type, with only one peak at around 70 nm ranging from 50–100 nm.

Another important quantity to monitor is the alpha-particle flux, which the cells under investigation are exposed to. Therefore, several studies include solid-state nuclear track detectors (SSNTD) like CR-39 films (Page Moulding, Pershore Limited, Pershore, UK) (Fukuvi Chemical Industry Co., Ltd., Fukui, Japan) which are exposed in the same manner as their samples [64,69,74]. During the exposure, alpha-particles strike these detectors and damage the polymer structure, which can be visualized using chemical etching followed by examination under the microscope to count the emerging tracks. The authors calculated the dose to the cells using the track density F (track/µm^2^) on CR-39 films, together with a calculated LET value of 139 keV/μm for alpha-particles emitted by ^222^Rn and a value that takes the geometrical parameters into account, calculated using the SRIM program [69]. The dose is obtained using the following formula:

D=0.16 LET × F

Interestingly, Hamza et al. claim that the same dosimetric approach was not feasible in their setups [74]. The CR-39 films and additionally LR-115 were used, as well as another SSNTD to measure the number of alpha tracks. However, inserting the films into the exposure bottle and blood samples caused damage to the film, which hindered the activity estimations.

As a last step in the dosimetry methodology, the measured quantities are used as input for radiation dose calculations, which relies on different assumptions depending on the measurement techniques that were used. Schumann et al. assumed that all alpha- and beta-particles were deposited locally and neglected any contribution from gamma-irradiation [75]. In follow-up studies, using the same experimental setup, only the alpha-dose was considered, which accounted for more than 96% of the total calculated dose [79,80]. Similarly, Hamza et al. calculated the dose applying the Marinelli formula originally derived for therapy [74,81]:

$$D=\frac{A\times T_{24}\times T_{eff}}{25\times m}$$

using the dose (*D*), the activity (*A*), the uptake in 24 h (*T*_24_), the effective half-life within the sample (*T_eff_*), the sample’s mass (*m*) as well as a unit conversion coefficient with a value of 25. However, the authors assumed uniform irradiation and only considered the alpha-particle contribution to the radiation dose. Jostes et al. also distinguished between activity attached to the cells and activity from the culture medium by using the liquid scintillation counter and NaI detector described above [60]. The activity associated with the cells was further considered to be uniformly distributed within the nucleus and cytoplasm of the cells.

### 2.4. Radiobiological Findings from Mammalian Cell Experiments with Radon Exposure Setups

Assuming the different measurement devices and dose calculations yield correct dose values, the biological results can be quantified and linked to the exposure conditions and respective radiation dose. As previously described, it is necessary to emphasize that the exposure of biological samples to radon and its progeny consists of a mixed radiation exposure, including alpha-particles, beta-particles and gamma-rays.

In order to investigate the clonogenic ability of cells after exposure to radon and progeny, colony survival assays can be used, which are based on the quantification of colonies generated by cells which maintained their proliferative capacity after radiation exposure [82]. 

An exponential decrease in clonogenic cell survival with increasing dose was observed for Chinese Hamster Ovary (CHO) cells by Jostes et al., independent of the different irradiation setups they used [60]. In this specific study, the CHO cell line was exposed in suspension to three different alpha-particle radiation sources (^212^Bi, a low activity radium source of 3 mCi and a 0.7 Ci ^226^RaCl_2_ source) at room temperature and plated for cell survival analysis after exposure. Depending on the radiation source, the authors reported very similar D_0_ values ranging from 61 ± 9 cGy to 67 ± 5 cGy for the different radiation sources. Therefore, they concluded that their simplest system using ^212^Bi could be used as a model of radon exposure to cells. 

A few years later, Chaudhry et al. also found an exponential dose-dependent decrease in clonogenic survival rates by investigating cytotoxic effects of radon exposures in human TK6 lymphoblasts by using a 2.96 Gbq ^226^RaCl_2_ source [83]. This study used the same setup as Bakale et al., which has been described above and in Table 1 [73]. Here, 250 kVp X-rays were used as reference radiation quality, resulting in a D_0_ of 63 cGy for X-rays compared to 29 cGy for radon and radon daughters.

The study of Petitot et al. quantified the portion of viable rat epithelial cells after exposure to radon and progeny via trypan blue staining, reporting a decrease in living cells compared to controls with time from 3 to 6 h [64]. Furthermore, Ding et al. evaluated the apoptosis level in HPBLs exposed to radon gas, emanating from a ^226^Rn source described in their previous study [69], by performing TUNEL staining [66]. The percentages of apoptotic HPBLs were significantly increased with radon exposure time for 3–6 h, resulting in a cumulative dose of 3.4–5.8 mGy compared to the control group that was exposed to natural background levels of radon, with the maximum percentage of apoptotic HPBLs at about 22%. 

Consistently, Cui et al. observed an increased apoptotic rate together with a shortened cell cycle after repeated radon exposure (10×) in transfected human bronchial cells (BEAS2B) using an Annexin-V/Propidium Iodide (PI) apoptosis assay [67]. However, culturing these cells for an additional 20 generations decreased the rate of apoptosis, which was accompanied by decreased contact inhibition [67]. 

Opposing results were found by Deloch et al., who also analyzed the apoptotic and necrotic rate using Annexin-V/PI staining in monocytes, macrophages and bone marrow isolated from mice [70]. After radon exposure, no significant changes in apoptosis were observed when compared to sham-irradiated control samples. 

Using the exposure setup by Cui et al., an increase in the proliferation and migration ability of human epithelial cells (16HBE, BEAS2B) was detected by Chen et al., 2020, accompanied by a reduction in cell adhesion after exposure to radon and progeny [67,84]. These characteristics also appear during epithelial–mesenchymal transition (EMT), which is known to promote carcinogenesis [85]. Studying this in further detail, the researchers discovered an upregulation of p-mTor, p-PI3K, p-AKT, β-catenin and GSK-3β [84]. Together with the obtained downregulation of E-cadherine and upregulation of Vimentin, FN1, α-SMA, N-Cadherin and Snail proteins, which serve as markers for EMT, the authors concluded that radon exposure could stimulate the PI3K/AKT/mTor-pathway, thus inducing EMT. 

In an earlier study, Cui et al. examined the up- and downregulation of regulatory microRNA (miRNA) in radon-exposed immortalized human lung epithelial cells (BEAS2B) compared to controls [67]. Detailed computational analysis of the regulated miRNAs indicated that pathways associated with cell proliferation and differentiations were stimulated. Furthermore, pathways for cell apoptosis, cell cycle, immune reactions and DNA repair were inhibited. The authors concluded that the differentially expressed miRNA target genes that were specifically up- or downregulated by radon exposure could be involved in the progression of tumorigenesis after radon exposure. 

By investigating the long term exposure of BEAS2B cells and human non-small cell lung cancer cells (NCI-H1975), Loiselle et al. also identified several differentially expressed genes during the experimental period that covered different time points over several months, which were mainly involved in regulation of cell signaling, cell growth and proliferation [68]. 

In addition to the previously described biological endpoints, alpha-particles can cause severe and complex DNA damage, of which DNA double-strand breaks (DSBs) are considered the most severe and lethal to irradiated cells. Once DNA DSBs are induced, a mammalian cell will activate a large pool of complementary proteins which are involved in the DNA damage response. Immunofluorescent staining of these proteins is frequently used to quantify DNA DSBs. 

One factor for early DNA DSB recognition, and subsequently for initiating repair mechanisms, is the MRE11-RAD50-NBS1 (MRN) sensor protein complex [86,87]. In a next step, the serine/threonine kinase ataxia telangiectasia mutated (ATM) is activated by an autophosphorylation (p-ATM) and recruited to the damage site, as well as a DNA-dependent protein kinase catalytic subunit (DNA-PKcs). Activated ATM phosphorylates different targets, such as the histone H2AX to γ-H2AX, the heterochromatin protein KAP-1 (p-KAP-1) and the p53 binding protein 1 (53BP1) [86,88,89]. Altogether, these proteins (γ-H2AX, 53BP1, pKAP-1, MRE11, p-ATM) are present at the DNA DSB site and are consequently used as markers to quantify radiation-induced DNA DSBs. 

Ding et al. and Wang et al. showed that exposure of HPBL to radon and progeny on a membrane, as described by Wang et al. [69], led to a dose-dependent linear increase in γ-H2AX, p-ATM, p-DNA-PKcs, 53BP1 and pKAP-1 foci and co-localizing foci tracks, with γ-H2AX being the most common signal [69]. However, these findings were independent of the radon activity concentration during exposure and interpreted by the authors as a lacking dose-rate effect. The analysis of the temporal change in foci and tracks after irradiation with a low radiation dose, estimated by the authors at 5.56 mGy, revealed an increase at 3 h post exposure compared to directly (0 h) fixed cells in the HPBLs. In the following hours (6 h, 9 h and 12 h), the foci and tracks decreased again but remained higher than background levels [66]. 

A dose-dependent increase in alpha-tracks and co-localization of γ-H2AX, 53BP1 and MRE 11 was also observed by another group in blood samples at 1 h after exposure to an absorbed dose of 100 mGy in a ^223^RaCl_2_ solution [75,80]. Göring et al. evaluated alpha-tracks by detecting γ-H2AX foci tracks in peripheral blood mononuclear cells occurring directly, 4 h and 24 h after exposure, showing a dose-dependent increase in DNA DSBs at the early time point [79]. The authors reported contradictory results compared to Ding et al., as average foci tracks after direct fixation were significantly higher than at later time points for all doses ≥ 25 mGy, and this trend was also seen with an absorbed dose of 3 mGy. Fixation at 24 h after irradiation showed that around 90% of alpha-particle-induced damage was repaired [79]. An additional investigation of the different protein frequencies present within tracks compared to the whole nucleus revealed that γ-H2AX was mainly present within the track, while less than 30% of 53BP1, MRE11 and pATM signals were within tracks [80]. 

It is important to note that the reported low radiation doses in these studies on DNA DSB detection and repair are referring to the whole sample dose. However, some (sub-) cellular regions will receive much higher local doses upon alpha-particle traversal. Again, the heterogeneous spatial dose distribution of radon exposure complicates our understanding of the observed low dose radiation effects, since they are the result of a mix of low and locally very high dose depositions in the in vitro cell cultures under investigation [90].

Not only direct DNA damage can be induced by ionizing radiation, but also chromatid and chromosome aberrations as a result of misrepair. A dose-dependent increase in chromosome aberrations after exposure to radon and progeny was observed in different studies. In addition, several studies also investigated the overdispersion of chromosomal aberrations, which is typical for high-LET radiation, since the probability that multiple aberrations will be generated with one particle traversal increases with LET [91]. At low and moderate doses of low-LET radiation, the distribution of aberrations can be well described by Poisson statistics, reflecting a simple random distribution of damages as expected according to the homogeneous pattern of energy depositions. In contrast, for experiments with alpha-particles, the spatial energy deposition is very inhomogeneous and concentrated along the particle track. Therefore, the variance on the distribution of the number of aberrations per cell exceeds unity and results in a greater variability in the data than would be expected based on a random Poisson distribution, resulting in overdispersion. This overdispersion describes an increased variance (*Var*(*X*)) compared to the expected value (*E*(*X*)) in the chosen statistical model, as one characteristic of the Poisson distribution is the equality of these two characteristics.

$$E\left(X\right)=Var\left(X\right)=\mu ;\;\mu \;\epsilon {\;R}_{+}$$

Wolff et al. found a dose-dependent as well as time-dependent increase in chromatid deletions in irradiated HPBLs and Hamza et al. confirmed this with analysis of dicentric chromosomal aberrations in blood samples [72,74]. Additionally, chromatid deletions also revealed an over dispersed Poisson distribution [72], similar to results obtained in immunofluorescent staining [66].

In their early study, Khan et al. obtained a dose-dependent increase in micronuclei in primary lung fibroblasts from rats, which was higher in the dividing fraction of cells (cell population >50% in S or G2/M phase) compared to non-dividing cells (cell population was 92.5% in G0/G1) at the time point of analysis [92]. This dose dependency was confirmed by Hamza et al. with the additional finding that micronuclei statistically showed an over dispersed Poisson distribution, which the same group also reported in their earlier study using the dicentric chromosome assay [74,93]. 

Jostes et al. compared the different types of mutations observed in CHO cells within the hypoxanthine phosphoribosyltransferase (HPRT) locus occurring spontaneously and after irradiation with low (0.25–0.30 Gy) and higher doses of radon and progeny (0.75–0.77 Gy) [94]. While the highest fraction of controls did not show any changes (74%), only a minor fraction revealed gene deletions and alterations. In contrast to that, gene deletions were the most commonly observed lesions in the radon irradiated samples. The observed differences within irradiated samples were not significant and therefore no effect of the higher dose could be identified. The authors stated that the calculated probability of multiple alpha-particle hits per cell is about a factor of two higher with the higher dose. Therefore, they raised the question whether cells which are hit by more than one alpha-particle might not be able to survive and are therefore not being detected with the used method, resulting in the lack of a dose-dependent effect. 

Chaudhry et al. analyzed the mutational pattern of lesions of the thymidine kinase (TK) gene in radon-exposed TK6 lymphoblasts compared to controls [83]. While the loss of heterozygosity extended over all analyzed loci (five genes) in 30% of the spontaneous mutations, it was only seen in 5% of radon exposed cells. However, 61% of radon-induced mutants showed a loss of heterozygosity in the TK gene and/or the two neighboring genes, compared to only 5% in controls. Therefore, radon most likely affects the TK gene and direct neighbors, representing a smaller more focused lesion, while the extended loss of genes seems rare after radon exposure [83].

**Table 1:** Summary of experimental studies on in vitro irradiation of mammalian cells with radon and its progeny.

Authors	Irradiation Setup	Dosimetry	Radon Source (Activity)	Radon Activity Concentration and/or Dose Rate	Additional Studies Performed with the Setup
Setups for adherent cell exposures					
Petitot et al. [64]	Exposure of adherent cells in chamber (10 m^3^) with humidity and CO_2_-concentration regulation and thermo-regulated bath inside Exposure in six-well plates on membrane with direct contact of cells with radon	Radon activity concentration: RAD 7 monitor Progeny collection on filter together with 3 independent alpha-particle counts Aerosol concentration + size distribution: Diffusion battery with condensation nuclei counter Alpha-particle flux: CR-39	^238^U + ^232^Th in underground tanks (uranothorianite)	c(Rn): 45–50 MBq/m^3^	
Cui et al. [67]	Exposure of adherent cells in chamber in water bath with continuously pumped radon gas through chamber Expsoure in Transwell^®^ plates and medium removed above cells before exposure	Not reported	RaCl_2_ source (25.9 GBq)	c(Rn): 20 kBq/m^3^	[84]
Maier et al. [65]	Exposure of adherent cells in water-jacketed chamber (50 L) with humidity and CO_2_-concentration regulation	Radon activity concentration: RTM 1688-2	^226^Ra source	Possible c(Rn): 0–620 kBq/m^3^	[70]
Wang et al. [69]	Cell monolayer exposure in Transwell^®^ plates Exposure chamber within water-jacketed incubator with saturated humidity	Radon activity concentration: AB5 radiation monitor Alpha-particle flux: CR-39 Dose calculation using track density and calculated LET (using SRIM program)	^226^Ra source (135 kBq)	c(Rn): 1000 kBq/m^3^	[66]
Loiselle et al. [68]	Long term exposure of adherent cells in culture plates with lid on inside incubator together with radon rock source	Radon activity concentration: AB5 radiation monitor	5 kg of batholith hematite granite (Standard 59033)	c(Rn): 38 Bq/m^3^	
Setups for irradiation of suspension cell cultures					
Jostes et al. [60]	Exposure of cells in suspension elution of ^212^Bi with HI solution, pH adjustment to 5 and addition of cells	Radon activity concentration: Ls 5801 + NaI detector Solving the Bateman-equation data used to calculate the activity of progeny and respectively ratios between radon and its progeny Measurement of activity associated with the cells compared to medium for dose calculations Assumption: activity associated with cells considered to be distributed uniformly	cation-exchange column containing ^224^Ra	Dose: 925 MBq	[92,94]
	Exposure of cells in suspension radon gas bubbled through culture medium, after 4–18 h addition of cells		^226^Ra source (111 MBq)	Dose rate: 3–8 cGy/h	
	Exposure of cells in suspension radon gas passed over surface of culture medium while continuously stirring it, cells added afterwards		^226^RaCl_2_ source (25.9 GBq)	Dose rate: 25–45 cGy/h	
Wolff et al. [72]	Exposure of cells in suspension within water-jacketed spinner flask Prior to cell addition via injection, radon gas is drawn over the medium	Scintillation spectrometry analogous to Jostes et al., 1991	^226^RaCl_2_ source (25.9 GBq)	Dose: 18 cGy	
Bakale et al. [73]	Exposure of cells in suspension in spinner flask System with source containment vessel, a pump and 2 syringes used to pump radon into syringe, after the injection of radon air into spinner flask	Radon activity concentration: NaI detector Calculation of activity of progeny analogous to Jostes et al., 1991	^226^Ra source (2.9 GBq)	Dose rates: 3–4 cGy/h	[83]
Hamza et al. [74]	Exposure of cells in suspension within glass bottle System with 3-way-valve to connect syringe, radon source and exposure bottle After radon injection: bottles on roller platform inside incubator	Radon activity concentration: Lucas cell Alpha-particle flux: CR-39 + LR-115 Dose calculation using Marinelli-formula	^226^Ra source (98.9 kBq)	c(Rn): 122 kBq/m^3^–1 593 MBq/m^3^ Dose: 0.01–127 mGy Dose rate: 0.000054–0.708 mGy/min	[93]
Schumann et al. [75]	Exposure of cells in suspension with ^223^Ra-dichloride solution by adding the solution to the sample after addition incubated on roller-mixer (35 rpm)	Measurement of radon activity concentration: HPGe detector assumption: alpha- and beta-particles deposited locally, contribution from gamma-rays neglected	^223^Ra-dichloride solution	c(Rn) in sample: 0.40–9.13 kBq/mL Dose: 0–136 mGy Dose coefficient: 16.1 mGy/kBq (Alphas: 15.5 mGy/kBq; Betas: 0.6 mGy/kBq)	[79,80]

## 3. In Vitro Studies on Mammalian Cells Exposed to Radon Analogues

Instead of the radon chambers described in the previous section, other isotopes that emit alpha-particles can be used for in vitro studies. These setups provide several advantages, such as an improved dose uniformity, an increase in the hit probability and an overall simplified experimental setup compared to the radon chambers [95]. It is recommended to select isotopes with a reasonable half-life and availability to mimic radon exposure, which avoids the need to permanently adjust the dosimetry due to the rapidly changing source activity, and it also reduces the costs. 

Commonly used isotopes for cell irradiations which emit comparable alpha-energies to radon are americium-241 (^241^Am) [96] and plutonium-238 (^238^Pu) [97], but plutonium-239 (^239^Pu) [98] or uranium-234 (^234^U) [99] have also been used. These isotopes, together with their respective decay energy, are summarized in Table 2. Alpha-decay results in the spontaneous emission of alpha-particles with energies between 4 and 6 MeV, which corresponds to high-LET values of approximately 108 keV/µm in water and up to 123 keV/µm in air [99]. 

As previously described, a reliable dose estimation is of crucial importance to all radiobiological experiments and includes knowledge of the LET, the fluence of the alpha-particles and the density of the target. The latter can be done either through measurements or using simulations, such as Monte Carlo simulations [95]. Furthermore, the dose uniformity in alpha-particle exposure systems is normally only achieved within limited ranges of source-to-target distances and cell dish sizes, which is an important point to take into consideration in the design of radon analogue setups [100]. The short distance between the source and target cells is crucial due to the short range of alpha-particles in the air, which is approximately 42 mm for ^241^Am alpha-particles [101]. 

Table 3 provides an overview of several radiobiology studies which used one of the four isotopes to expose mammalian cells to radon analogues. 

### 3.1. Plutonium-239 (^239^Pu)

Although most setups irradiate cells in a monolayer attached to a thin foil, Purrott et al. used a different approach by mixing a plutonium citrate solution with a blood sample to investigate the yield of unstable chromosome aberrations in lymphocytes [98]. They found a linear relation between the probability of dicentric aberrations and the radiation dose in the range of 0.13–1.6 Gy. Dosimetry was performed by measuring the plutonium content of the blood solution, which might give rise to large uncertainties as there will be a variety of different energies of the alpha-particles and thus the LET and dose. 

In all following exposure setups, cells were grown in a monolayer on thin Mylar-foils and irradiated in close proximity to an external alpha-source. 

### 3.2. Uranium-234 (^234^U)

Nikitaki et al. described a ^234^U source (total activity of 0.77 ± 0.03 MBq) where the small setup can be placed in a vapor-saturated incubator, which protects the cells from drying out and provides the possibility for long irradiation times and intervals [99]. The alpha-particles emitted in the air have a mean energy of 4.9 MeV at the surface of a thin disk deposited on an aluminum substrate. Source surface homogeneity (<10–15%) and dosimetry have been validated by measurements and Monte Carlo simulations under various environmental conditions. The optimal distance between the source and cells appeared to be 14.8 mm in this setup. In order to validate the setup biologically, the authors evaluated the yield of chromosomal aberrations (dicentric chromosomes and centric rings) in CHO cells, which resulted in 0.25 ± 0.03 aberrations at 24 h post-irradiation to 13 min 24 s, corresponding to an estimated radiation dose of 0.57 Gy. The number of chromosomal aberrations was significantly elevated compared to sham-irradiated control samples. 

Furthermore, immunofluorescence staining for γ-H2AX and 53BP1 was performed in CHO cells and resulted in 3.26 ± 1.28 foci/100 µm^2^, corresponding to 0.78 Gy. The biological validation corresponded well with previously published values and simulations, which made the authors conclude that their setup is usable for in vitro experiments with mammalian cells at low doses and low dose rates.

### 3.3. Plutonium-238 (^238^Pu)

The ^238^Pu irradiator established by Goodhead et al. has been in use since 1983 [97]. It allows irradiation with well-defined alpha-energy (3.26 MeV), LET (121 keV/µm), direction, dose and dose rate. Except for the direction, all the other parameters can be varied by adjusting the distance of the source to the cells. For dosimetric purposes, energy spectra and fluence measurements were performed with a surface barrier Si detector and CR-39 detectors, respectively. This setup was used in subsequent years for a large variety of radiobiological experiments. In one of these studies, it was shown that the ability of V79-4 cells from the lung of a male Chinese hamster to rejoin double strand breaks after alpha-particle irradiation is significantly reduced compared to gamma-radiation from a ^60^Co-source [102]. 

In another study, the proportion and complexity of chromosomal aberrations increased after 0.5 Gy of alpha-particle irradiation in the HPBLs of four different donors, compared to 3 Gy of low-LET reference X-ray irradiation [103]. The complex aberrations involving three or more breaks in two or more chromosomes were evaluated, resulting in 49–56% complex aberrations relative to total exchanges for alpha-particle irradiation, compared to 20–22% for X-rays. Additionally, 15% of the complex aberrations induced by alpha-particles at the first division were potentially transmissible, and by the third division transmissible-type complex aberrations such as insertions represented 65% of all complex type aberrations [103]. These types of stable aberrations could potentially persist through many cell generations. 

A similar approach was investigated by Themis et al. for human bronchial lung epithelial cells from two donors. They found an increased frequency and complexity of complex chromosomal aberrations at approximately 1 alpha-particle/nucleus, yet only 1–2% of all damage was transmissible to future cell generations [104]. 

Another more recent study investigated the mechanism of recognition and processing of complex DNA damage in HeLa and oropharyngeal squamous cell carcinoma cells. They found the Histone H2B ubiquitylated on lysine 120 is induced several hours after irradiation by alpha-particles, a histone post-translational modification which is important in the promotion of chromatin relaxation and the recruitment of DNA damage repair proteins [46]. It is important to mention that despite the fact that the authors mention 3.26 MeV alpha-particles with an LET of 121 keV/µm produced by a ^238^Pu irradiator, they do not directly refer to the setup designed by Goodhead et al. in their paper [97]. However, since a clear description of the alpha-particle irradiation setup was lacking, we took the freedom to assume that the previously described ^238^Pu irradiator was used for these experiments.

Ultimately, Tracy et al. investigated the RBE for cell survival in V79-4 cells as a function of the energy of the alpha-particle. In order to enable these experiments, the ^238^Pu alpha-particle irradiator was calibrated for incident energies ranging from 4.0 MeV down to 1.1 MeV. They found an increasing RBE with a maximum RBE_10%_ (at 10% survival) of 4.49 ± 0.07 or RBE_M_ (maximum low-dose RBE or alpha ratio) of 10.2 ± 0.2 for 3.2 MeV alpha-particles, corresponding to an average LET of 131 keV/μm [105]. 

Hakanen et al. built an irradiation setup that was inspired by that of Goodhead et al. with only minor changes. The distance between the source and cells can be varied, which subsequently allows the user to vary the LET and particle energy. Measured alpha-spectra were compared to Monte Carlo simulations in order to confirm dosimetry [106].

The setup of Tisnek et al. again follows a similar approach as the one by Goodhead et al., with a ^238^Pu source at various distances to the exit window. The source is operational under vacuum. Dosimetry was done by alpha-spectroscopic measurements and Monte Carlo simulations with dose rates between 14.5 and 1.2 Gy/s, depending on the distance [107].

Simmons, Cohn and Min used a different setup with a ^238^Pu source. The cells were irradiated at a fixed distance on a rotating source to ensure homogeneous irradiation. Dosimetry was done by using track film badges for fluence measurements and known stopping power values from published tables. The yield of chromosomal aberrations in V79 hamster cells and human lung cells was found to be linearly related with dose, resulting in a D_0_ of 0.78 and 0.37 Gy for hamster and human cells, respectively [108].

Lastly, Metting et al. followed a similar concept with their ^238^Pu source, where the distance between cells and the source was fixed [109]. Dosimetry was performed using CR-39 detectors to determine fluence, source homogeneity and residual range. The corresponding LET values were calculated with respective software. For this approach, a dose rate of 0.1 Gy/min was determined.

### 3.4. Americium-241 (^241^Am)

The most commonly used isotope for in vitro radon analogue irradiations is ^241^Am, as it has the same alpha-energy of ^222^Rn of 5.49 MeV. Beaton et al. developed such a system at Health Canada (Ottawa) which can be placed inside an incubator during irradiation and makes use of Mylar-based culture dishes [101]. Dosimetry was performed using GEANT4 Monte Carlo simulations, taking into account the geometry and physical properties. The average dose rate was calculated to be 0.98 ± 0.01 Gy/h and the LET 127.4 ± 0.04 keV/µm. For validation, these results were compared to survival curves obtained for human lung epithelial cells (A549), resulting in an RBE of 6.3. 

This setup was also used for an investigation on the changes in apoptosis and gene expression after alpha-particle and X-ray irradiation [110]. They found that, on average, 33% of human monocytic cells (THP-1 cell line) were apoptotic at 1.5 Gy of alpha-particle radiation at four days post-irradiation compared to <5% with X-ray irradiation. In addition, transcript profiling showed that Tumor necrosis factor alpha (TNF-α) and Fas Cell Surface Death Receptor (Fas) were commonly upregulated after three different doses of alpha-particle radiation, suggesting that the signaling cascades of these two genes might be involved in radiation-induced cell death after alpha-particle irradiation. 

Roos and Kellerer developed an irradiation device with an attached collimator to decrease the energy variability of the emitted alpha-particle. Additionally, a mechanical shutter was located between the source and the target cells [111]. This shutter was shielding the alpha-particles, and by adjusting the opening time, the dose could be adjusted. The chromosome aberrations in HPBLs was found to have a linear increase with a yield of 0.27 dicentrics per cell and per Gy, and resulted in an RBE_M_ of 15 [112]. Here, the authors stimulated separated lymphocytes with phytohaemagglutinin (PHA) in dishes consisting of a stainless steel or glass ring of 5 cm in diameter and a Mylar bottom foil of 2 µm and allowed them to attach for 3 h. Thereafter, the medium was removed and cells were washed with cell culture medium to remove any unattached cells, so a cell monolayer could be exposed to the alpha-particles emitted by the ^241^Am source.

A similar approach was implemented by Wang and Coderre, where an ^241^Am source is placed in a holder and cells are separated by a mechanical shutter from the sources [113]. There are several sources available with activities ranging from 0.37 to 3700 kBq. Dosimetry was done by measuring the energy spectra of the alpha-particles at the position of the cells and the corresponding LET values were calculated using simulation software, resulting in an average alpha-particle energy of 3.14 MeV and an LET of 128 keV/µm. The fluence was measured using CR-39 track detectors. 

This approach is very similar to the ^241^Am alpha-particle irradiation setup established by Maier et al., using the same dosimetric approach and comparable setup, with the restriction that only a source with an activity of 25 MBq is available [95]. Additionally, plasma treated Mylar foil is used to increase the polarity and thus the wettability with polar liquids. 

The setup of Wang was used for the irradiation of human prostate carcinoma (DU-145) cells to investigate medium-mediated bystander effects [113]. An increase of approximately 50% in micronucleus formation was observed in non-target co-cultured cells, but the addition of DMSO to the medium during irradiation blocked the bystander effect, suggesting the involvement of short-lived radical species. However, a follow-up study showed no decrease in the surviving fraction and no increase in γ-H2AX foci induction in co-cultured bystander cells of either DU-145 or AG01522 human fibroblast cells [114]. 

At Seoul National University, another alpha irradiation setup for in vitro cell irradiation with an ^241^Am source was developed [100]. Depending on the necessary exposure rate, the source activity and the distance between the source and cell culture dish can be adjusted. The irradiation time is aligned by a mechanical shutter. For dosimetric purposes, the energy spectrum at the sample position was measured and the corresponding LET values were derived from the NIST (USA) homepage [115]. The irradiation system was equipped with cell dishes of 4 µm thick Mylar bottom and a specific setup for cells on slides was available for γ-H2AX foci assay. For biological validation, the γ-H2AX foci assay was used in order to validate the expected hits per cell, which was also done in the setup designed by Maier et al. [95]. 

In a later study, Lee and Kim investigated the toxicity and effects of alpha-particle exposure on human lung epithelial cells (BEAS-2B, Null-1) and mouse vascular endothelial cells (SVEC4-10EHR1) by performing γ-H2AX staining and a clonogenic survival assay [116]. All three cell lines showed a dose-dependent linear increase in γ-H2AX foci per cell, with SVEC4-10EHR1 being most sensitive to alpha-particles. Additionally, the clonogenic survival rates in SVEC4-10EHR1 and BEAS-2B cells decreased exponentially with increasing alpha-particle exposure.

At Queen’s University in Belfast, Moreira et al. developed an alpha irradiation setup where cell cultures were irradiated in Mylar dishes by placing them at different distances from an ^241^Am source. The irradiation time was manually adjusted by removing the cells from the source. Source uniformity checks by Gafchromic film measurements, fluence by CR-39 detectors and alpha-energy by alpha-spectroscopy were performed for dosimetry purposes. These data were benchmarked against Monte Carlo simulations [117].

**Table 3:** Summary of experimental studies on in vitro irradiation of mammalian cells with radon analogues.

Authors	Irradiation Setup	Dosimetry	Source Activity	Dose/Dose Rate	Additional Studies Performed with the Setup
Plutonium-239 (^239^Pu)					
Purrott et al. [98]	Exposure of cells in suspension with ^239^Pu nitrate and ^241^Am nitrate solution; samples were gently agitated during irradiation	Measurement of plutonium content in the sample by scintillation counting, americium content by measuring X-ray emission		Variable	
Uranium-234 (^234^U)					
Nikitaki et al. [99]	Exposure of adherent cells growing on Mylar foil; setup can be placed in an incubator	Measurement for source homogeneity, MC simulations for LET and energy deposition	0.77 ± 0.03 MBq		
Plutonium-238 (^238^Pu)					
Goodhead et al. [97]	Exposure of adherent cells growing on Mylar foil; distance between source and cell culture can be varied	Measurement of energy spectra with surface barrier Si detector, fluence measurement with CR-39	1.2 GBq	Variable Maximum: 23.4 Gy/min	[46,102,103,104,105]
Metting et al. [109]	Exposure of adherent cells growing on Mylar foil; fixed distance between source and sample	Measurement of fluence and homogeneity with CR-39 detectors; calculation of LET with respective software	296 MBq	0.1 mGy/min	
Simmons et al. [108]	Exposure of adherent cells growing on Mylar foil; fixed distance between source and sample; source was rotated to allow uniform irradiation	Fluence measurements with neutron track film badges, LET data from literature			
Hakanen et al. [106]	Exposure of adherent cells growing on Mylar foil; adapted setup from Goodhead et al.; distance between source and cell culture can be varied	Measurement of energy spectra with high-resolution spectrometer; validation by MC-simulations	0.93 ± 0.04 GBq	Variable	
Tisnek et al. [107]	Exposure of adherent cells growing on Mylar foil; adapted setup from Goodhead et al.; distance between source and cell culture can be varied	Measurement of energy spectra with high-resolution spectrometer; validation by MC-simulations	1.3 ± 0.1 GBq	Variable	
Americium-241 (^241^Am)					
Roos & Kellerer [111]	Exposure of adherent cells growing on Mylar foil; fixed distance between source and sample; source was rotated to allow uniform irradiation	Measurement of energy spectra with semiconductor detector, calculation of fluence, LET and absorbed dose	0.37 GBq	0.2 Gy/min	[112]
Wang et al. [113]	Exposure of adherent cells growing on Mylar foil; fixed distance between source and sample	Measurement of energy spectra with semiconductor detector, measurement of fluence with CR-39; calculation of LET with respective software	0.37–3700 kBq	Variable	[114]
Beaton et al. [101]	Exposure of adherent cells growing on Mylar foil; setup can be placed in an incubator	MC-simulations	65 kBq	0.98 ± 0.01 Gy/h	[110]
Lee et al. [100]	Exposure of adherent cells growing on Mylar foil; variable distance between source and sample	Measurement of energy spectra with semiconductor detector, corresponding LET values were derived from a database	37 kBq 370 kBq 3.7 MBq	Variable	[116]
Maier et al. [95]	Exposure of adherent cells growing on plasma treated Mylar foil; fixed distance between source and sample	Measurement of energy spectra with semiconductor detector, measurement of fluence with CR-39; calculation of LET with respective software	25 MBq	8.2 ± 2.4 Gy/min	
Moreira et al. [117]	Exposure of adherent cells growing on Mylar foil; variable distance between source and sample	Measurement of source homogeneity with Gafchromic film, fluence with CR-39 detector and energy spectra with a semiconductor silicon detector	7.4 MBq	Variable	

## 4. Discussion

Understanding the health effects of radon exposure remains one of the most important topics in radiation protection, since radon exposure contributes to approximately 50% of the annual effective dose from natural background radiation and has been identified as a potent carcinogen and the second leading cause of lung cancer after cigarette smoking [1,118]. Despite epidemiological studies revealing a sub-multiplicative interaction of both factors [15,119], there are currently no in vitro studies combining both agents which could illustrate the synergistic effect of radon exposure and tobacco smoke. 

Particularly indoor radon, due to the accumulation of radon gas in confined spaces such as homes and workplaces, can pose a significant health risk [120,121]. A better understanding of the underlying biological mechanisms giving rise to this increased cancer risk can be obtained through experimental studies where mammalian cells are exposed to radon and its progeny, or isotopes emitting alpha-particles of a similar energy. Therefore, several research groups have developed radon and radon analogue exposure systems for in vitro experiments, which are summarized in Table 1 and Table 3.

One of the most important challenges in cancer risk assessments related to radon exposure is the uncertainty in radon dose determination. The latter is particularly complicated due to the complexity and heterogeneity of the dose distribution upon radon exposure, also referred to as a spatial variation in dose delivery [44]. This heterogeneous component is present at different scales, starting with the lung at the organism level, which is the most exposed and affected organ when radon is inhaled [90]. The deposition of the radon progeny, and thereby the dose, is heterogeneous and characterized by high dose depositions in small groups of cells, located in hotspots such as the bronchial airway bifurcations [122,123]. Together with the variable thickness of lung mucus, this means that only a few cells will receive a very high dose and a majority of cells will receive no dose at all [42]. In addition, there is the synergistic interaction of cigarette smoking with indoor radon progeny, which has been evaluated in modelling studies [124,125]. The association of radon progeny with larger particles in cigarette smoke results in the selective deposition in hot spots at bronchial bifurcations.

Furthermore, the highly localized energy deposition of alpha-particle tracks, together with their limited range, results in a pronounced inhomogeneity of energy deposition at the subcellular level. As a result, the energy deposition in the traversed cell nuclei is determined by the probability of alpha-particle hits and the energy deposited in the case of a hit. The latter is directly linked with the biological effects that will be observed at the cellular level. 

Several microdosimetric models have been developed over the years to relate a specific energy distribution at the (sub)cellular level to radiobiological effects, which have been reviewed in detail in previous review papers. [126,127]. Especially for studying the impact of this inhomogeneous dose distribution and to simplify the exposure conditions compared to in vivo experiments, in vitro studies with mammalian cell lines can be extremely useful. As described by Madas et al., this approach can provide valuable input and validation for mathematical models for cancer risk assessments [44]. 

However, also for in vitro studies, selecting the right dosimetric approach remains a critical component to conducting a successful experiment. The most important parameters for dose determination are the fluence and the LET of the traversing alpha-particle. This can be measured with a great variety of detectors such as silicon surface barrier detectors for energy measurements or CR-39 detectors for fluence measurements. These results can further be validated using MC simulations or software for LET calculation like SRIM. Due to their short range, there will always be a spectrum of different particle energies which needs to be taken into account that can result in a great source of uncertainty. The situation gets more complicated for irradiation with radon and its progeny compared to radon analogues. 

For exposure of cells in suspension, as well as for adherent cells which are covered with medium, pure radon gas first needs to dissolve in the medium and will subsequently decay at a random location. The same will happen for the radon progeny, which have different decay energies. Therefore, the cells will be irradiated with a very broad energy (or LET spectrum) causing huge dose uncertainties. The most common measurable parameter for radon is the activity concentration, which can be done with commercially available detector systems. To obtain the dose, dose conversion factors are usually used, which are discussed intensely [128]. Hence, radon analogues provide the advantage of a better characterized energy spectrum and thus more reliable dosimetry.

One could argue that one of the disadvantages of the in vitro exposure setups is that they usually emit a mixed field of different radiation qualities. Therefore, it could be questioned if these radiobiological findings might be compromised by a gamma component in some setups, compared to experiments where irradiation was performed with pure alpha-particles such as accelerator produced helium beams. However, the mixed radiation field is much closer to what happens in reality when an individual inhales radon gas. To the best of our knowledge, the dose contribution of the gamma component that is emitted by sources that are commonly used for alpha-particle irradiations is approximately a factor 1000 lower than the radiation dose delivered by the alpha component itself [111]. Therefore, this gamma component is usually neglected in the final dose calculation. Nevertheless, it should be noted that the beta and gamma component can make up for around 10% of the decay energy [26]. As they have a larger range in matter, the probability of a cell getting hit by one of those irradiation types is further enhanced, increasing their dose contribution.

In addition to the heterogeneous variation in the radon dose deposition, there is also the effect of the temporal variation in the case of protracted, low-dose radon exposure. Some studies support an inverse exposure-rate effect, while others report minimal effects of the dose rate of alpha-particle irradiation on the shape of a cell survival curve [129]. The argument used by Beaton et al. for the large difference in dose rate between the reference ^137^Cs gamma irradiation (49 ± 3 Gy/h) and their ^241^Am source (0.98 ± 0.01 Gy/h) [101] is based on the idea that the majority of damage is caused by complex DNA DSBs. Lowering the dose rate will have little effect on the cell survival, since the complexity of the DNA damage that is created by the alpha-particle tracks will not change [130]. 

The traversal of a single alpha-particle through a cell nucleus becomes extremely relevant in the case of low-dose radon exposures, since only a few cells in the tissue will be hit by an alpha-particle, but one of those hits can already be sufficient to induce complex DNA damage and a potential lethal lesion [131,132]. The comparison of the two survival curves by Beaton et al. resulted in an RBE of 6.3 ± 3.0 for 10% survival for A549 cells [101], which is within the range of other RBE values reported in literature for 10% survival with alpha-particle radiation. For example, 9.9 for 10% survival for ^210^Po irradiated bovine endothelial cells [133] and approximately 3.8 ± 0.4 at 10% survival for different fibroblast cell lines [134]. 

However, the concept of RBE is deeply rooted in radiotherapy and based on reproductive cell death measurements, where one aims to achieve tumor control. Therefore, one could question if this concept of RBE is applicable to radiation protection scenarios where cancer risks are evaluated, which are the results of genomic instability and mutations in cells that, on the contrary, survive the radiation exposure. The dose rate is an important point to consider in the design of radon chambers or radon analogue setups if one aims to mimic an in vivo protracted, low dose radon exposure. For radon exposure related to natural background radiation, the dose rate is about 2 mSv/year, which can be enhanced by approximately one order of magnitude when living in an area with high radon levels. By using radon analogues, the dose rates are usually in the mGy per hour or day region, which is much higher than background levels.

It is well known that the repair of DNA DSBs is crucial to prevent chromosomal instability or cell death [135]. In this context, it is important to note that alpha-particle-induced DNA DSBs are repaired slower than DSBs induced by sparsely low-LET ionizing radiation, which has been observed with the setup of Nikitaki et al. [99]. 

As expected, several groups also reported an increase in DNA DSBs in HPBLs using different radon exposure systems compared to sham-irradiated control samples, even at very low doses (<10 mGy) [66,79]. The lowest cumulative absorbed dose that could be estimated by Ding et al. was 1.74 mGy based on the linear dose response curve obtained for γ-H2AX foci [66]. However, there seems to be a contradiction in the timing of these effects, since most studies report the highest level of γ-H2AX foci or DNA DSBs formation in the first 0.5 h post exposure, while Ding et al. report a delayed effect where the highest level of γ-H2AX foci was observed at 3 h and remained significantly increased in the first 6 h post exposure to a dose of 5.56 mGy [66]. In addition, the yield of linear tracks of the three different DNA repair proteins (γ-H2AX, 53BP1 and pKAP-s) remained constant in the first 3 h. According to the authors, this may be explained by the dissolution of radon gas in the cell culture medium and the resulting deposition of radon progeny, leading to a sustained internal alpha-particle irradiation. 

In the more recent study of Göring et al., the HPBLs were exposed to a ^223^RaCl_2_ solution and irradiated with doses of 3, 25, 50 and 100 mGy [75,79], which are higher than the doses used by Ding et al. [66]. Interestingly, the results of Göring et al. suggest that the induction as well as the repair rate of the DNA DSBs were dependent on the absorbed dose to the blood, which could potentially also explain the difference in the γ-H2AX foci peak between the studies of the different groups [79]. 

The biological effects of radon exposure are not restricted to the cells that were directly traversed by an alpha-particle, but also in neighboring cells, which are described as non-targeted or bystander effects. These effects seem to be not linearly related with dose and appear at very low dose levels [136,137]. This was also observed in the study of Wang et al., where human prostate cancer cells were co-incubated for 2 h on an insert in a co-culture system above irradiated cells exposed to alpha-particle doses ranging from 0.1 up to 6.0 Gy and their irradiated medium. All conditions caused a similar level of micronucleus formation in the non-targeted cells, independent of the radiation dose [113]. 

The effect seems to be mediated by the production of reactive oxygen species and the presence of the irradiated cells, since the bystander effects disappeared when the irradiated cells were not present during the 2 h co-incubation or when DMSO was added to the medium during irradiation. However, contradicting results have been reported for in vitro studies with radon exposure setups so far and some authors even questioned the relevance of alpha-particle-induced bystander effects in domestic radon risk estimations [138]. Furthermore, many elements which seem to be fundamental to bystander signaling, such as the dependence on gap-junction communication, could vary with cell type [137]. 

The latter invites for a discussion on the selection of a relevant cell line for radon and radon analogue studies, since large variations exist in the intrinsic radiosensitivity and RBE values reported for different cell lines [139]. One of the reasons for this variation is the difference in DNA damage repair capacity between different cell types and also factors like the cell cycle phase play a role [140,141]. 

Again, in vitro experiments offer a relatively easy and straightforward way to study the DNA repair capacity and the competition between different DNA repair pathways that are involved in the repair of the complex DNA damage induced by alpha-particles. However, due to the large variation in the radiosensitivity of cells, it is advisable to select cell types which are most probably the target cells for cancer initiation after radon exposure, such as the basal and secretory cells of the bronchial epithelium [50,142]. Approximately one third of the studies listed in Table 1 and Table 3 use cells of lung origin, of which a large fraction are the well-known Chinese hamster lung fibroblast cell line V-79 together with lung fibroblasts from rat origins [64,92,102,105,108]. However, some studies worked with BEAS-2B or other lung epithelial cell lines as well [67,68,84,104,116]. 

The use of Chinese hamster cell lines, such as V79 or CHO cells [99], is not surprising when a new radon or radon analogue exposure setup needs to be validated. These cell lines are commonly used in radiobiology experiments, so they are well characterized and can be easily compared to previously published studies on the same radiobiological endpoints with other radiation qualities. In addition, these cells lines are immortalized and known to have exponential growth patterns, which makes them much easier to culture and handle in experiments compared to primary cell lines. 

However, if one aims to go beyond initial validation experiments, it is important to select a cell line of relevance to the pre- and neoplastic transformation which might be induced by radon exposure. Furthermore, there are a handful of studies which used malignant, immortalized cell lines for their in vitro experiments with radon analogues [46,101,113,114,117] and a large proportion of studies worked with blood or bone marrow cells [60,70,72,74,75,79,80,93,97,98,103,110,112]. The latter is relevant to investigate the effect of radon exposure on the immune system, which could have repercussions on the development of hypersensitivity and increase susceptibility to infections and lung cancer [143]. 

Furthermore, these studies are also very interesting in the context of radon treatments for chronic painful inflammatory diseases, which remains a controversial application [22,70]. In addition to the radiation protection and clinical applications, blood cells and particularly HPBLs are commonly used in radiobiology studies since they can be easily sampled for biodosimetry studies and circulating lymphocytes are a synchronic non-cycling population in G0/G1, which makes it easier to rule out and control cell cycle effects [144].

Next to the variation in inherent radiosensitivity and repair capacity of cells, which was previously described, it is clear that the cellular environment of the exposed cells plays an important role in the ultimately observed health effects. Especially in the case of inhomogeneous radon exposures, the tissue environment might have a substantial impact on the induced biological effects. Recently, there have been developments in 3D cultures for human bronchial epithelial cells and organ-on-chip technologies which provide opportunities to use in vitro model systems to study the effect of inhomogeneous radon exposure in a physiologically relevant cell environment [145]. Several generation of lung-on-chip models have been developed so far and it will be interesting to see how these intercellular interactions and DNA repair processes are affected by radon exposure in these in vivo like conditions [146,147].

Furthermore, another source of valuable information to understand the complex effects of radon intake and the dose deposition of the radon progeny is through in vivo animal experiments. Histological findings of in vivo experiments provide the opportunity to identify hotspots for neoplastic changes and reflect the biological processes at organism level, where cancers are formed in a complex biological environment. 

In addition, studies on humans are necessary and are currently extremely scarce [26]. However, one of the main challenges in in vivo studies remains the inhomogeneous dose distribution, which makes it challenging to translate the biological observations to a radon exposure level. From this point of view, in vitro studies with radon chambers and radon analogues remain important to unravel the fundamental mechanisms and their dose response relationships, which could eventually give rise to genomic instability and cancer initiation.

## 5. Conclusions

In order to get a better understanding of the health effects induced by protracted low dose exposure to radon, we need to intensify and standardize in vitro and in vivo experiments which investigate the mechanisms involved in radon carcinogenesis. Despite multiple decades of radon research and its well-documented carcinogenic potential, the number of in vitro radiobiology studies remains scarce and often inconclusive. Unfortunately, conclusions are often confounded by dosimetric uncertainties, as well as a large variation in the applied radiation doses in in vitro cell experiments, dose rates and biological parameters. This review provides an overview of the experimental setups that have been used so far for in vitro radon research and highlights that factors such as the reliability of the dosimetry, the selection of cell lines and the exposure conditions (presence of culture media, temperature…) need to be critically considered during the design phase of the experiment. 

In vivo experiments using appropriate model systems or humans exposed to radon are finally essential to understand the complex mechanisms underlying radon exposure and resulting effects. However, for ethical reasons, not all in vivo studies that are required to answer the open questions can be done in humans. In addition, for mechanistic studies, focused and well designed in vitro experiments can usefully complement dedicated studies in animal models. Their results can provide opportunities to optimize current mathematical models, risk estimations and eventually contribute to appropriate cancer prevention strategies.

## Figures and Tables

**Table 2:** Isotopes used for in vitro irradiation with their respective decay energies, emission probabilities and half-life [63].

Isotope	Decay Energy Alpha Particle [MeV]	Emission Probability [%]	Half-Life [a]
^234^U	4.722	28.4	2.455 × 10^5^
	4.774	71.4	
^238^Pu	5.456	29.0	87.7
	5.499	70.9	
^239^Pu	5.106	11.5	2.411 × 10^4^
	5.144	15.1	
	5.157	73.3	
^241^Am	5.443	13.0	432.2
	5.486	84.5	

## Data Availability

No new data were created or analyzed in this study. Data sharing is not applicable to this article.

## References

[1] World Health Organization (2009). WHO Handbook on Indoor Radon: A Public Health Perspective.

[2] Vogiannis E.G., Nikolopoulos D. (2015). Radon Sources and Associated Risk in Terms of Exposure and Dose. Front. Public Health.

[3] ICRP (2017). Occupational Intakes of Radionuclides: Part 3. ICRP Publication 137. Ann. ICRP.

[4] Čeliković I., Pantelić G., Vukanac I., Nikolić J.K., Živanović M., Cinelli G., Gruber V., Baumann S., Poncela L.S.Q., Rabago D. (2022). Outdoor Radon as a Tool to Estimate Radon Priority Areas—A Literature Overview. Int. J. Environ. Res. Public Health.

[5] Harrison J.D., Marsh J.W. (2020). ICRP Recommendations on Radon. Ann. ICRP.

[6] Bouder F., Perko T., Lofstedt R., Renn O., Rossmann C., Hevey D., Siegrist M., Ringer W., Pölzl-Viol C., Dowdall A. (2019). The Potsdam Radon Communication Manifesto. J. Risk Res..

[7] Cori L., Curzio O., Donzelli G., Bustaffa E., Bianchi F. (2022). A Systematic Review of Radon Risk Perception, Awareness, and Knowledge: Risk Communication Options. Sustainability.

[8] Seltenrich N. (2019). Radon Risk: A Global Estimate of Radon’s Contribution to Lung Cancer. Environ. Health Perspect..

[9] Cinelli G., Tollefsen T., Bossew P., Gruber V., Bogucarskis K., De Felice L., De Cort M. (2019). Digital Version of the European Atlas of Natural Radiation. J. Environ. Radioact..

[10] Michael S., Andreas B., Claudia S., Fabian R., Ringer W., Josef M.F. (2014). Radon in Waterworks: Dose Assessment, Analysis of Influence Parameters and Improved Methods of Measurement. Radiat. Prot. Dosim..

[11] ICRP (2016). Occupational Intakes of Radionuclides: Part 2. ICRP Publication 134. Ann ICRP.

[12] Wiegand J. (2001). A Guideline for the Evaluation of the Soil Radon Potential Based on Geogenic and Anthropogenic Parameters. Environ. Geol..

[13] Craft B.F., Oser J.L. (2007). A Method for Determining Relative Amounts of Combined and Uncombined Radon Daughter Activity in Underground Uranium Mines. Am. Ind. Hyg. Assoc. J..

[14] Hunter N., Muirhead C.R., Tomasek L., Kreuzer M., Laurier D., Leuraud K., Schnelzer M., Grosche B., Placek V., Heribanova A. (2013). Joint Analysis of Three European Nested Case-Control Studies of Lung Cancer among Radon Exposed Miners: Exposure Restricted to below 300 WLM. Health Phys..

[15] Kreuzer M., Sobotzki C., Schnelzer M., Fenske N. (2018). Factors Modifying the Radon-Related Lung Cancer Risk at Low Exposures and Exposure Rates among German Uranium Miners. Radiat. Res..

[16] Tirmarche M., Harrison J., Laurier D., Blanchardon E., Paquet F., Marsh J. (2012). Risk of Lung Cancer from Radon Exposure: Contribution of Recently Published Studies of Uranium Miners. Ann. ICRP.

[17] IARC Working Group (1988). Man-Made Mineral Fibres and Radon.

[18] Darby S., Hill D., Auvinen A., Barros-Dios J.M., Baysson H., Bochicchio F., Deo H., Falk R., Forastiere F., Hakama M. (2005). Radon in Homes and Risk of Lung Cancer: Collaborative Analysis of Individual Data from 13 European Case-Control Studies. Br. Med. J..

[19] Krewski D., Lubin J.H., Zielinski J.M., Alavanja M., Catalan V.S., Field R.W., Klotz J.B., Létourneau E.G., Lynch C.F., Lyon J.L. (2006). A Combined Analysis of North American Case-Control Studies of Residential Radon and Lung Cancer. J. Toxicol. Environ. Health A.

[20] Menzler S., Piller G., Gruson M., Rosario A.S., Wichmann H.E., Kreienbrock L. (2008). Population Attributable Fraction for Lung Cancer Due to Residential Radon in Switzerland and Germany. Health Phys..

[21] Kreuzer M., Fenske N., Schnelzer M., Walsh L. (2015). Lung Cancer Risk at Low Radon Exposure Rates in German Uranium Miners. Br. J. Cancer.

[22] Maier A., Wiedemann J., Rapp F., Papenfuß F., Rödel F., Hehlgans S., Gaipl U.S., Kraft G., Fournier C., Frey B. (2020). Radon Exposure-Therapeutic Effect and Cancer Risk. Int. J. Mol. Sci..

[23] Cheng E.S., Egger S., Hughes S., Weber M., Steinberg J., Rahman B., Worth H., Ruano-Ravina A., Rawstorne P., Yu X.Q. (2021). Systematic Review and Meta-Analysis of Residential Radon and Lung Cancer in Never-Smokers. Eur. Respir. Rev..

[24] Ngoc L.T.N., Park D., Lee Y.C. (2022). Human Health Impacts of Residential Radon Exposure: Updated Systematic Review and Meta-Analysis of Case-Control Studies. Int. J. Environ. Res. Public Health.

[25] Mozzoni P., Pinelli S., Corradi M., Ranzieri S., Cavallo D., Poli D. (2021). Environmental/Occupational Exposure to Radon and Non-Pulmonary Neoplasm Risk: A Review of Epidemiologic Evidence. Int. J. Environ. Res. Public Health.

[26] Papenfuß F., Maier A., Fournier C., Kraft G., Friedrich T. (2022). In-Vivo Dose Determination in a Human after Radon Exposure: Proof of Principle. Radiat. Environ. Biophys..

[27] Bräuner E.V., Loft S., Sørensen M., Jensen A., Andersen C.E., Ulbak K., Hertel O., Pedersen C., Tjønneland A., Kjær S.K. (2015). Residential Radon Exposure and Skin Cancer Incidence in a Prospective Danish Cohort. PLoS ONE.

[28] Ruano-Ravina A., Aragonés N., Kelsey K.T., Pérez-Ríos M., Piñeiro-Lamas M., López-Abente G., Barros-Dios J.M. (2017). Residential Radon Exposure and Brain Cancer: An Ecological Study in a Radon Prone Area (Galicia, Spain). Sci. Rep..

[29] Harley N.H., Robbins E.S. (2009). Radon and Leukemia in the Danish Study: Another Source of Dose. Health Phys..

[30] Henshaw D.L., Eatough J.P., Richardson R.B. (1990). Radon as a Causative Factor in Induction of Myeloid Leukaemia and Other Cancers. Lancet.

[31] Moon J., Yoo H.K. (2021). Residential Radon Exposure and Leukemia: A Meta-Analysis and Dose-Response Meta-Analyses for Ecological, Case-Control, and Cohort Studies. Environ. Res..

[32] Barbosa-Lorenzo R., Barros-Dios J.M., Ruano-Ravina A. (2017). Radon and Stomach Cancer. Int. J. Epidemiol..

[33] Eatough J.P., Henshaw D.L. (1992). Radon and Thoron Associated Dose to the Basal Layer of the Skin. Phys. Med. Biol..

[34] Stather J.W. (2004). Dosimetric and Epidemiological Approaches to Assessing Radon Doses--Can the Differences Be Reconciled?. Radiat. Prot. Dosim..

[35] Marsh J.W., Harrison J.D., Laurier D., Blanchardon E., Paquet F., Tirmarche M. (2010). Dose Conversion Factors for Radon: Recent Developments. Health Phys..

[36] Porstendorfer J., Reineking A. (1992). Indoor Behaviour and Characteristics of Radon Progeny. Radiat. Prot. Dosim..

[37] Knutson E.O., Tu K.W. (1996). Size Distribution of Radon Progeny Aerosol in the Working Area of a Dry Former Uranium Mine. Environ. Int..

[38] Steck D.J., Field R.W. (2006). Dosimetric Challenges for Residential Radon Epidemiology. J. Toxicol. Environ. Health A.

[39] Shimo M., Ikebe Y. (1984). Measurements of Radon and Its Short-Lived Decay Products and Unattached Fraction in Air. Radiat. Prot. Dosim..

[40] Sakoda A., Ishimori Y., Yamaoka K., Kataoka T., Mitsunobu F. (2013). Absorbed Doses of Lungs from Radon Retained in Airway Lumens of Mice and Rats. Radiat. Environ. Biophys..

[41] Füri P., Farkas Á., Madas B.G., Hofmann W., Winkler-Heil R., Kudela G., Balásházy I. (2020). The Degree of Inhomogeneity of the Absorbed Cell Nucleus Doses in the Bronchial Region of the Human Respiratory Tract. Radiat. Environ. Biophys..

[42] Madas B.G., Drozsdik E.J. (2018). Effects of Mucus Thickness and Goblet Cell Hyperplasia on Microdosimetric Quantities Characterizing the Bronchial Epithelium upon Radon Exposure. Int. J. Radiat. Biol..

[43] Markovic V.M., Stevanovic N., Nikezic D. (2011). Doses from Beta Radiation in Sensitive Layers of Human Lung and Dose Conversion Factors Due to 222Rn/ 220Rn Progeny. Radiat. Environ. Biophys..

[44] Madas B.G., Boei J., Fenske N., Hofmann W., Mezquita L. (2022). Effects of Spatial Variation in Dose Delivery: What Can We Learn from Radon-Related Lung Cancer Studies?. Radiat. Environ. Biophys..

[45] Hall E.J., Giaccia A.J. (2011). Radiobiology for the Radiologist.

[46] Carter R.J., Nickson C.M., Thompson J.M., Kacperek A., Hill M.A., Parsons J.L. (2018). Complex DNA Damage Induced by High Linear Energy Transfer Alpha-Particles and Protons Triggers a Specific Cellular DNA Damage Response. Int. J. Radiat. Oncol..

[47] James A.C., Birchall A., Akabani G. (2004). Comparative Dosimetry of BEIR VI Revisited. Radiat. Prot. Dosim..

[48] Sgouros G. (2008). Alpha-Particles for Targeted Therapy. Adv. Drug Deliv. Rev..

[49] Gaillard S., Pusset D., De Toledo S.M., Fromm M., Azzam E.I. (2009). Propagation Distance of the Alpha-Particle-Induced Bystander Effect: The Role of Nuclear Traversal and Gap Junction Communication. Radiat. Res..

[50] Szo’ke I., Farkas A., Balásházy I., Hofmann W., Madas B.G., Szőke R. (2012). 3D-Modelling of Radon-Induced Cellular Radiobiological Effects in Bronchial Airway Bifurcations: Direct versus Bystander Effects. Int. J. Radiat. Biol..

[51] De Toledo S.M., Buonanno M., Harris A.L., Azzam E.I. (2017). Genomic Instability Induced in Distant Progeny of Bystander Cells Depends on the Connexins Expressed in the Irradiated Cells. Int. J. Radiat. Biol..

[52] Azzam E.I., De Toledo S.M., Little J.B. (2003). Expression of CONNEXIN43 Is Highly Sensitive to Ionizing Radiation and Other Environmental Stresses. Cancer Res..

[53] Deshpande A., Goodwin E.H., Bailey S.M., Marrone B.L., Lehnert B.E. (1996). Alpha-Particle-Induced Sister Chromatid Exchange in Normal Human Lung Fibroblasts: Evidence for an Extranuclear Target. Radiat. Res..

[54] Abu Shqair A., Kim E.H. (2021). Multi-Scaled Monte Carlo Calculation for Radon-Induced Cellular Damage in the Bronchial Airway Epithelium. Sci. Rep..

[55] Sgouros G., Roeske J.C., Mcdevitt M.R., Palm S., Allen B.J., Fisher D.R., Brill A.B., Song H., Howell R.W., Akabani G. (2010). MIRD Pamphlet No. 22 (Abridged): Radiobiology and Dosimetry of a-Particle Emitters for Targeted Radionuclide Therapy* In Collaboration with the SNM MIRD Committee. J. Nucl. Med..

[56] Brenner D.J. (1992). Radon: Current Challenges in Cellular Radiobiology. Int. J. Radiat. Biol..

[57] Crawford-Brown D.J., Hofmann W. (1996). The Testing of Radiobiological Models of Radon Carcinogenesis Needed for in Vitro to in Vivo Extrapolations. Environ. Int..

[58] Robertson A., Allen J., Laney R., Curnow A. (2013). The Cellular and Molecular Carcinogenic Effects of Radon Exposure: A Review. Int. J. Mol. Sci..

[59] Riudavets M., Garcia de Herreros M., Besse B., Mezquita L. (2022). Radon and Lung Cancer: Current Trends and Future Perspectives. Cancers.

[60] Jostes R.F., Hui T.E., James A.C., Cross F.T., Schwartz J.L., Rotmensch J., Atcher R.W., Evans H.H., Mencl J., Bakale G. (1991). In Vitro Exposure of Mammalian Cells to Radon: Dosimetric Considerations. Radiat. Res..

[61] Hinrichs A., Schmitt M., Papenfuß F., Roth M., Fournier C., Hinrichs A., Schmitt M., Papenfuß F., Roth M., Fournier C. (2023). Radon Solubility in Different Tissues after Short Term Exposure. Int. J. Environ. Res. Public Health.

[62] Sanjon E.P., Maier A., Hinrichs A., Kraft G., Drossel B., Fournier C. (2019). A Combined Experimental and Theoretical Study of Radon Solubility in Fat and Water. Sci. Rep..

[63] NuDat 3. https://www.nndc.bnl.gov/nudat3/.

[64] Petitot F., Morlier J.P., Debroche M., Pineau J.F., Chevillard S. (2002). A New Method Specifically Designed to Expose Cells Isolated In Vitro to Radon and Its Decay Products. Radiat. Res..

[65] Maier A., van Beek P., Hellmund J., Durante M., Schardt D., Kraft G., Fournier C. (2015). Experimental Setup for Radon Exposure and First Diffusion Studies Using Gamma Spectroscopy. Nucl. Instrum. Methods Phys. Res. Sect. B Beam Interact. Mater. Atoms.

[66] Ding D., Zhang Y., Wang J., Wang X., Fan D., He L., Zhang X., Gao Y., Li Q., Chen H. (2016). γ-H2AX/53BP1/PKAP-1 Foci and Their Linear Tracks Induced by in Vitro Exposure to Radon and Its Progeny in Human Peripheral Blood Lymphocytes. Sci. Rep..

[67] Cui F.M., Li J.X., Chen Q., Du H.B., Zhang S.Y., Nie J.H., Cao J.P., Zhou P.K., Hei T.K., Tong J. (2013). Radon-Induced Alterations in Micro-RNA Expression Profiles in Transformed BEAS2B Cells. J. Toxicol. Environ. Health A.

[68] Loiselle J.J., Knee J.M., Sutherland L.C. (2019). Human Lung Epithelial Cells Cultured in the Presence of Radon-Emitting Rock Experience Gene Expression Changes Similar to Those Associated with Tobacco Smoke Exposure. J. Environ. Radioact..

[69] Wang J., He L., Fan D., Ding D., Wang X., Gao Y., Zhang X., Li Q., Chen H. (2016). Establishment of a γ-H2AX Foci-Based Assay to Determine Biological Dose of Radon to Red Bone Marrow in Rats. Sci. Rep..

[70] Deloch L., Hehlgans S., Rückert M., Maier A., Hinrichs A., Flohr A.S., Eckert D., Weissmann T., Seeling M., Nimmerjahn F. (2022). Radon Improves Clinical Response in an Animal Model of Rheumatoid Arthritis Accompanied by Increased Numbers of Peripheral Blood B Cells and Interleukin-5 Concentration. Cells.

[71] Atcher R.W., Friedman A.M., Hines J.J. (1988). An Improved Generator for the Production of 212Pb and 212Bi from 224Ra. Int. J. Rad. Appl. Instrum. A.

[72] Wolff S., Jostes R., Cross F.T., Hui T.E., Afzal V., Wiencke J.K. (1991). Adaptive Response of Human Lymphocytes for the Repair of Radon-Induced Chromosomal Damage. Mutat. Res..

[73] Bakale G., Rao P.S., Mencl J., Adams R.B., Evans H.H. (1993). A Radon Generator/Delivery System. Radiat. Res..

[74] Hamza Z.V., Kumar V.P.R., Jeevanram R.K., Santanam R., Danalaksmi B., Mohankumar M.N. (2008). A Simple Method to Irradiate Blood Cells in Vitro with Radon Gas. Radiat. Prot. Dosim..

[75] Schumann S., Eberlein U., Muhtadi R., Lassmann M., Scherthan H. (2018). DNA Damage in Leukocytes after Internal Ex-Vivo Irradiation of Blood with the α-Emitter Ra-223. Sci. Rep..

[76] Bateman H. (1910). The Solution of a System of Differential Equations Occuring in the Theory of Radioactive Transformations. Proc. Camb. Philos. Soc. Math. Phys. Sci..

[77] Thomas J.W. (1970). Modification of the Tsivoglou Method for Radon Daughters in Air. Health Phys..

[78] Tsivoglou E., Ayer H., Holaday D. (1953). Occurence of Nonequilibrium Atmospheric Mixtures of Radon and Its Daughters. Nucleon. Ceased Publ..

[79] Göring L., Schumann S., Müller J., Buck A.K., Port M., Lassmann M., Scherthan H., Eberlein U. (2022). Repair of α-Particle-Induced DNA Damage in Peripheral Blood Mononuclear Cells after Internal Ex Vivo Irradiation with 223Ra. Eur. J. Nucl. Med. Mol. Imaging.

[80] Scherthan H., Lee J.H., Maus E., Schumann S., Muhtadi R., Chojowski R., Port M., Lassmann M., Bestvater F., Hausmann M. (2019). Nanostructure of Clustered DNA Damage in Leukocytes after In-Solution Irradiation with the Alpha Emitter Ra-223. Cancers.

[81] Marinelli L.D. (1949). Dosage Determination in the Use of Radioactive Isotopes. J. Clin. Investig..

[82] Franken N.A.P., Rodermond H.M., Stap J., Haveman J., van Bree C. (2006). Clonogenic Assay of Cells in Vitro. Nat. Protoc..

[83] Chaudhry M.A., Jiang Q., Ricanati M., Horng M.F., Evans H.H. (1996). Characterization of Multilocus Lesions in Human Cells Exposed to X Radiation and Radon. Radiat. Res..

[84] Chen H., Chen N., Li F., Sun L., Du J., Chen Y., Cheng F., Li Y., Tian S., Jiang Q. (2020). Repeated Radon Exposure Induced Lung Injury and Epithelial-Mesenchymal Transition through the PI3K/AKT/MTOR Pathway in Human Bronchial Epithelial Cells and Mice. Toxicol. Lett..

[85] Thiery J.P., Acloque H., Huang R.Y.J., Nieto M.A. (2009). Epithelial-Mesenchymal Transitions in Development and Disease. Cell.

[86] McCarthy-Leo C., Darwiche F., Tainsky M.A. (2022). DNA Repair Mechanisms, Protein Interactions and Therapeutic Targeting of the MRN Complex. Cancers.

[87] Qiu S., Huang J. (2021). MRN Complex Is an Essential Effector of DNA Damage Repair. J. Zhejiang Univ. Sci. B.

[88] White D., Rafalska-Metcalf I.U., Ivanov A.V., Corsinotti A., Peng H., Lee S.C., Trono D., Janicki S.M., Rauscher F.J. (2012). The ATM Substrate KAP1 Controls DNA Repair in Heterochromatin: Regulation by HP1 Proteins and Serine 473/824 Phosphorylation. Mol. Cancer Res..

[89] Dahl E.S., Aird K.M. (2017). Ataxia-Telangiectasia Mutated Modulation of Carbon Metabolism in Cancer. Front. Oncol..

[90] Madas B.G. (2016). Radon Exposure and the Definition of Low Doses-The Problem of Spatial Dose Distribution. Health Phys..

[91] Virsik R.P., Harder D. (1981). Statistical Interpretation of the Overdispersed Distribution of Radiation-Induced Dicentric Chromosome Aberrations at High LET. Radiat. Res..

[92] Khan M.A., Cross F.T., Jostes R., Hui E., Morris J.E., Brooks A.L. (1994). Micronuclei Induced by Radon and Its Progeny in Deep-Lung Fibroblasts of Rats In Vivo and In Vitro. Radiat. Res..

[93] Hamza V.Z., Mohankumar M.N. (2009). Cytogenetic Damage in Human Blood Lymphocytes Exposed in Vitro to Radon. Mutat. Res. Mol. Mech. Mutagen..

[94] Jostes R.F., Fleck E.W., Morgan T.L., Stiegler G.L., Cross F.T. (1994). Southern Blot and Polymerase Chain Reaction Exon Analyses of HPRT- Mutations Induced by Radon and Radon Progeny. Radiat. Res..

[95] Maier A., Wiedemann J., Adrian J.A., Dornhecker M., Zipf A., Kraft-Weyrather W., Kraft G., Richter S., Teuscher N., Fournier C. (2019). α-Irradiation Setup for Primary Human Cell Cultures. Int. J. Radiat. Biol..

[96] Franken N.A.P., Hovingh S., Ten Cate R., Krawczyk P., Stap J., Hoebe R., Aten J., Barendsen G.W. (2012). Relative Biological Effectiveness of High Linear Energy Transfer α-Particles for the Induction of DNA-Double-Strand Breaks, Chromosome Aberrations and Reproductive Cell Death in SW-1573 Lung Tumour Cells. Oncol. Rep..

[97] Goodhead D.T., Bance D.A., Stretch A., Wilkinson R.E. (1991). A Versatile Plutonium-238 Irradiator for Radiobiological Studies with Alpha-Particles. Int. J. Radiat. Biol..

[98] Purrott R.J., Edwards A.A., Lloyd D.C., Stather J.W. (1980). The Induction of Chromosome Aberrations in Human Lymphocytes by in Vitro Irradiation with Alpha-Particles from Plutonium-239. Int. J. Radiat. Biol. Relat. Stud. Phys. Chem. Med..

[99] Nikitaki Z., Choulilitsa E., Kalospyros S.A., Kaisaridi S., Terzoudi G.I., Kokkoris M., Georgakilas A.G. (2021). Construction and Evaluation of an α-Particle-Irradiation Exposure Apparatus. Int. J. Radiat. Biol..

[100] Lee K.M., Lee U.S., Kim E.H. (2016). A Practical Alpha Particle Irradiator for Studying Internal Alpha Particle Exposure. Appl. Radiat. Isot..

[101] Beaton L.A., Burn T.A., Stocki T.J., Chauhan V., Wilkins R.C. (2011). Development and Characterization of an in Vitro Alpha Radiation Exposure System. Phys. Med. Biol..

[102] Jenner T.J., DeLara C.M., O’Neill P., Stevens D.L. (1993). Induction and Rejoining of DNA Double-Strand Breaks in V79-4 Mammalian Cells Following Gamma- and Alpha-Irradiation. Int. J. Radiat. Biol..

[103] Anderson R.M., Marsden S.J., Wright E.G., Kadhim M.A., Goodhead D.T., Griffin C.S. (2009). Complex Chromosome Aberrations in Peripheral Blood Lymphocytes as a Potential Biomarker of Exposure to High-LET Alpha-Particles. Int. J. Radiat. Biol..

[104] Themis M., Garimberti E., Hill M.A., Anderson R.M. (2013). Reduced Chromosome Aberration Complexity in Normal Human Bronchial Epithelial Cells Exposed to Low-LET γ-rays and High-LET α-Particles. Int. J. Radiat. Biol..

[105] Tracy B.L., Stevens D.L., Goodhead D.T., Hill M.A. (2015). Variation in RBE for Survival of V79-4 Cells as a Function of Alpha-Particle (Helium Ion) Energy. Radiat. Res..

[106] Hakanen A., Siiskonen T., Pöllänen R., Kosunen A., Turunen A., Belyakov O. (2006). Design, Spectrum Measurements and Simulations for a 238Pu α-Particle Irradiator for Bystander Effect and Genomic Instability Experiments. Appl. Radiat. Isot..

[107] Tisnek N., Kalanxhi E., Serkland C.W., Iversen J., Belyakov O.V., Dahle J. (2009). A 238Pu Irradiator for Exposure of Cultured Cells with Alpha-Radiation: Construction, Calibration and Dosimetry. Appl. Radiat. Isot..

[108] Simmons J.A., Cohn P., Min T. (1996). Survival and Yields of Chromosome Aberrations in Hamster and Human Lung Cells Irradiated by Alpha Particles. Radiat. Res..

[109] Metting N.F., Koehler A.M., Nagasawa H., Nelson J.M., Little J.B. (1995). Design of a Benchtop Alpha Particle Irradiator. Health Phys..

[110] Chauhan V., Howland M., Chen J., Kutzner B., Wilkins R.C. (2011). Differential Effects of Alpha-Particle Radiation and X-Irradiation on Genes Associated with Apoptosis. Radiol. Res. Pract..

[111] Roos H., Kellerer A.M. (1989). Design Criteria and Performance Parameters of an Alpha Irradiation Device for Cell Studies. Phys. Med. Biol..

[112] Schmid E., Hieber L., Heinzmann U., Roos H., Kellerer A.M. (1996). Analysis of Chromosome Aberrations in Human Peripheral Lymphocytes Induced by in Vitro α-Particle Irradiation. Radiat. Environ. Biophys..

[113] Wang R., Coderre J.A. (2005). A Bystander Effect in Alpha-Particle Irradiations of Human Prostate Tumor Cells. Radiat. Res..

[114] Anzenberg V., Chandiramani S., Coderre J.A. (2008). LET-Dependent Bystander Effects Caused by Irradiation of Human Prostate Carcinoma Cells with X rays or Alpha Particles. Radiat. Res..

[115] U.S. Department of Commerce National Institute of Standards and Technology. http://www.nist.gov.

[116] Lee U.S., Kim E.H. (2019). Combined Effect of Alpha Particles and Cigarette Smoke on Human Lung Epithelial Cells in Vitro. Int. J. Radiat. Biol..

[117] Moreira H.M., Guerra Liberal F.D., McMahon S.J., Prise K.M. (2021). Characterization of a Custom-Made 241Am Alpha-Source for Radiobiological Studies. Appl. Radiat. Isot..

[118] United Nations Scientific Committee on the Effects of Atomic Radiation (2017). Sources, Effects and Risks of Ionizing Radiation.

[119] Lubin J.H. (1988). Models for the Analysis of Radon-Exposed Populations. Yale J. Biol. Med..

[120] United Nations Scientific Committee on the Effects of Atomic Radiation (2009). Effects of Ionizing Radiation.

[121] Kim J., Farré M., Auvil L., Capitanu B., Larkin D.M., Ma J., Lewin H.A. (2017). Reconstruction and Evolutionary History of Eutherian Chromosomes. Proc. Natl. Acad. Sci. USA.

[122] Balásházy I., Hofmann W. (2000). Quantification of Local Deposition Patterns of Inhaled Radon Decay Products in Human Bronchial Airway Bifurcations. Health Phys..

[123] Hofmann W., Crawford-Brown D.J., Menache M.G., Martonen T.B. (1991). Carcinogenic Risk of Non-Uniform Alpha Particle Irradiation in the Lungs: Radon Progeny Effects at Bronchial Bifurcations. Radiat. Prot. Dosim..

[124] Martonen T.B., Hofmann W. (1991). Dosimetry of Localised Accumulations of Cigarette Smoke and Radon Progeny at Bifurcations. Radiat. Prot. Dosim..

[125] Martell E.A. (1983). Alpha-Radiation Dose at Bronchial Bifurcations of Smokers from Indoor Exposure to Radon Progeny. Proc. Natl. Acad. Sci. USA.

[126] Hofmann W., Li W.B., Friedland W., Miller B.W., Madas B., Bardiès M., Balásházy I. (2020). Internal Microdosimetry of Alpha-Emitting Radionuclides. Radiat. Environ. Biophys..

[127] Baiocco G., Bartzsch S., Conte V., Friedrich T., Jakob B., Tartas A., Villagrasa C., Prise K.M. (2022). A Matter of Space: How the Spatial Heterogeneity in Energy Deposition Determines the Biological Outcome of Radiation Exposure. Radiat. Environ. Biophys..

[128] Mc Laughlin J.P. (2019). Dosimetric and epidemiological approaches to radon lung cancer risk assessment. Radiat. Prot. Dosim..

[129] Lowe D., Roy L., Tabocchini M.A., Rühm W., Wakeford R., Woloschak G.E., Laurier D. (2022). Radiation Dose Rate Effects: What Is New and What Is Needed?. Radiat. Environ. Biophys..

[130] Claesson A.K., Stenerlöw B., Jacobsson L., Elmroth K. (2007). Relative Biological Effectiveness of the Alpha-Particle Emitter (211)At for Double-Strand Break Induction in Human Fibroblasts. Radiat. Res..

[131] Timm S., Lorat Y., Jakob B., Taucher-Scholz G., Rübe C.E. (2018). Clustered DNA Damage Concentrated in Particle Trajectories Causes Persistent Large-Scale Rearrangements in Chromatin Architecture. Radiother. Oncol..

[132] Lorat Y., Timm S., Jakob B., Taucher-Scholz G., Rübe C.E. (2016). Clustered Double-Strand Breaks in Heterochromatin Perturb DNA Repair after High Linear Energy Transfer Irradiation. Radiother. Oncol..

[133] Thomas P., Tracy B., Ping T., Baweja A., Wickstrom M., Sidhu N., Hiebert L. (2007). Relative Biological Effectiveness (RBE) of Alpha Radiation in Cultured Porcine Aortic Endothelial Cells. Int. J. Radiat. Biol..

[134] Raju M.R., Eisen Y., Carpenter S., Inkret W.C. (1991). Radiobiology of α Particles. III. Cell Inactivation by α-Particle Traversals of the Cell Nucleus. Radiat. Res..

[135] Cannan W.J., Pederson D.S. (2016). Mechanisms and Consequences of Double-Strand DNA Break Formation in Chromatin. J. Cell. Physiol..

[136] Azzam E.I., Little J.B. (2016). The Radiation-Induced Bystander Effect: Evidence and Significance. Hum. Exp. Toxicol..

[137] Blyth B.J., Sykes P.J. (2011). Radiation-Induced Bystander Effects: What Are They, and How Relevant Are They to Human Radiation Exposures?. Radiat. Res..

[138] Brenner D.J., Sachs R.K. (2003). Domestic Radon Risks May Be Dominated by Bystander Effects--but the Risks Are Unlikely to Be Greater than We Thought. Health Phys..

[139] Flint D.B., Bright S.J., McFadden C.H., Konishi T., Ohsawa D., Turner B., Lin S.H., Grosshans D.R., Chiu H.S., Sumazin P. (2021). Cell Lines of the Same Anatomic Site and Histologic Type Show Large Variability in Intrinsic Radiosensitivity and Relative Biological Effectiveness to Protons and Carbon Ions. Med. Phys..

[140] Weyrather W.K., Ritter S., Scholz M., Kraft G. (1999). RBE for Carbon Track-Segment Irradiation in Cell Lines of Differing Repair Capacity. Int. J. Radiat. Biol..

[141] Shrivastav M., De Haro L.P., Nickoloff J.A. (2008). Regulation of DNA Double-Strand Break Repair Pathway Choice. Cell Res..

[142] Leach J.P., Morrisey E.E. (2018). Repairing the Lungs One Breath at a Time: How Dedicated or Facultative Are You?. Genes Dev..

[143] Nagarkatti M., Nagarkatti P.S., Brooks A. (1996). Effect of Radon on the Immune System: Alterations in the Cellularity and Functions of T Cells in Lymphoid Organs of Mouse. J. Toxicol. Environ. Health.

[144] Inghirami G., Zhu B.Y., Chess L., Knowles D.M. (1990). Flow Cytometric and Immunohistochemical Characterization of the Gamma/Delta T-Lymphocyte Population in Normal Human Lymphoid Tissue and Peripheral Blood. Am. J. Pathol..

[145] Rayner R.E., Makena P., Prasad G.L., Cormet-Boyaka E. (2019). Optimization of Normal Human Bronchial Epithelial (NHBE) Cell 3D Cultures for in Vitro Lung Model Studies. Sci. Rep..

[146] Hiemstra P.S., Grootaers G., van der Does A.M., Krul C.A.M., Kooter I.M. (2018). Human Lung Epithelial Cell Cultures for Analysis of Inhaled Toxicants: Lessons Learned and Future Directions. Toxicol. In Vitro.

[147] Zamprogno P., Wüthrich S., Achenbach S., Thoma G., Stucki J.D., Hobi N., Schneider-Daum N., Lehr C.M., Huwer H., Geiser T. (2021). Second-Generation Lung-on-a-Chip with an Array of Stretchable Alveoli Made with a Biological Membrane. Commun. Biol..

